# Single‐Cell RNA Sequencing Uncovers Pathological Processes and Crucial Targets for Vascular Endothelial Injury in Diabetic Hearts

**DOI:** 10.1002/advs.202405543

**Published:** 2024-10-30

**Authors:** Yan Zhang, Yang Cao, Xuebin Zhang, Jie Lin, Mengyuan Jiang, Xiao Zhang, Xinchun Dai, Xiaohua Zhang, Yue Liu, Wen Ge, Huanhuan Qiang, Congye Li, Dongdong Sun

**Affiliations:** ^1^ Department of Cardiology Xijing Hospital Fourth Military Medical University Xi'an 710032 China

**Keywords:** cardiovascular, diabetic injury, endothelial cells, single‐cell analysis

## Abstract

Cardiovascular disease remains the leading cause of high mortality in individuals with diabetes mellitus. Endothelial injury is a major contributing factor for vascular dysfunction in diabetes. However, the precise mechanisms underlying endothelial cell injury and their heterogeneity in diabetes remains elusive. In this study, single‐cell sequencing is performed in heart tissues from leptin receptor knock‐out (db/db) diabetic mice at various pathological stages. Through cell cluster identification, differential gene analysis, intercellular communication analysis, pseudo time analysis, and transcription factor analysis, a novel mechanism of cardiac vascular endothelial damage in diabetes is identified. Specifically, a single‐cell transcription map of cardiac vascular endothelial cells is presented in db/db mice. Diverse cellular clusters are found to play vital roles under diabetes‐induced damage, highlighting crucial transcription factors involved in their regulation. In addition, the essential transcription factor Ets1 is found to protect against vascular endothelial injury in db/db mice. In summary, the work provides a comprehensive understanding of the development of diabetic cardiac vascular endothelial damage and the heterogeneity of the cells involved. These findings offer valuable insights into potential treatments and assessments of diabetic cardiovascular endothelial damage.

## Graphical abstract

This work provides a comprehensive understanding of the development of diabetic cardiac vascular endothelial damage and the heterogeneity of the cells involved. These findings offer valuable insights into potential treatments and assessments of diabetic cardiovascular endothelial damage.

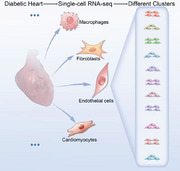

## 1 Introduction

According to a recent epidemiological survey by the International Diabetes Federation (IDF), ≈536 million adults worldwide were estimated to be afflicted with diabetes in 2021, with this number projected to surpass 700 million by 2045.^[^ ^1^ ^]^ The alarming prevalence of diabetes not only imposes a heavy medical burden but also ranks as a leading cause of death globally,^[^ ^2^ ^]^ emphasizing the pertinent need to better manage diabetes and its related complications.

It is well understood that both type 1 and type 2 diabetes have a detrimental impact on blood vessels in multiple organs.^[^ ^3^, ^4^ ^]^ Cardiovascular anomalies remain the primary cause of mortality in diabetic individuals. Conversely, the abrupt rise in the prevalence of diabetes is also a major contributing factor to the high incidence of cardiovascular diseases.^[^ ^5^, ^6^ ^]^ Cardiac tissues contain a rich vascular network, and vascular dysfunction often manifests as an early symptom in diabetes patients.^[^ ^7^ ^]^ Early stages of vascular anomalies gradually develop into irreversible structural disorders. This progression mainly involves vascular rarefaction and adverse remodeling of small arteries, resulting in impaired diastolic and systolic function of the heart.^[^ ^8^ ^]^

Endothelial cells (ECs) are the predominant non‐myocytes in the heart.^[^ ^9^ ^]^ ECs respond to shear stress imposed by blood flow through regulation of the thickness and diameter of blood vessel walls while controlling the entry of immune cells and molecules into sub‐endothelial tissues.^[^ ^10^, ^11^ ^]^ To accommodate the diverse needs of the vascular system, ECs typically consist of heterogeneous clusters with distinct functions.^[^ ^12^, ^13^ ^]^

Endothelial injury is the leading cause of vascular dysfunction in diabetes mellitus, characterized by increased secretion of vasoconstrictive factors, reduced cell viability, elevated endothelial permeability, compromised nitric oxide (NO) production, and thrombosis.^[^ ^14^, ^15^ ^]^ In cases of early exposure to elevated blood glucose levels, ECs suffer persistent damage, even under tightly controlled blood glucose conditions.^[^ ^16^, ^17^ ^]^ However, the heterogeneity of ECs in diabetic injury and the underlying mechanism(s) remains unclear, hindering effective prevention and treatment of diabetic cardiovascular injury.

Single‐cell sequencing enables the identification of diverse cell types and their gene expression at the level of individual cells, providing unique insights into biological development and disease progression.^[^ ^18^, ^19^ ^]^ This technique reveals cellular sequence discrepancies in specific microenvironments, offering an accurate representation of cell heterogeneity within given samples. In our study, we used leptin receptor knockout (db/db) mice as diabetic models and sequenced hearts of these mice at different pathological stages. Through cell cluster identification, differential gene analysis, intercellular communication analysis, pseudo time analysis, and transcription factor analysis, we were able to unveil essential mechanisms of diabetic cardiac vascular endothelial injury.

## 2 Results

### 2.1 Characterization of Cardiac Vascular Endothelial Cells Heterogeneity

To systematically describe the pathological vascular endothelial injury process in diabetic hearts, single‐cell sequencing was conducted in db/db mice at different stages of diabetic injury. To mitigate the impact of leptin receptors on sequencing outcomes, db/m mice pairs were chosen as controls rather than wild‐type (WT) mice. We monitored blood glucose levels and observed that they were elevated at 6 weeks and stabilized at 8 weeks (>20mmol L^−1^) in db/db mice. Furthermore, db/db mice started to die at 20 weeks of age. In this context, we used 8‐week db/db mice (db/8w) as an early stage of diabetic impairment, with age‐matched heterozygous mice (db/m) as the control group. The 20‐week‐old db/db (db/20w) mice were classified as late‐stage, whereas 14‐week‐old db/db (db/14w) mice were considered as middle‐stage. Additionally, we assessed the cardiac functionality and structural modifications of the mice in each group prior to sequencing. We observed that, with progression, the cardiac function of db/db mice progressively declined (Figure S1A,B, Supporting Information), perivascular fibrosis exacerbated (Figure S1C,D, Supporting Information), and cardiomyocytes progressively dilated (Figure S1E,F, Supporting Information), confirming that our model mice were at distinct stages of diabetic injury.

**Figure 1:**
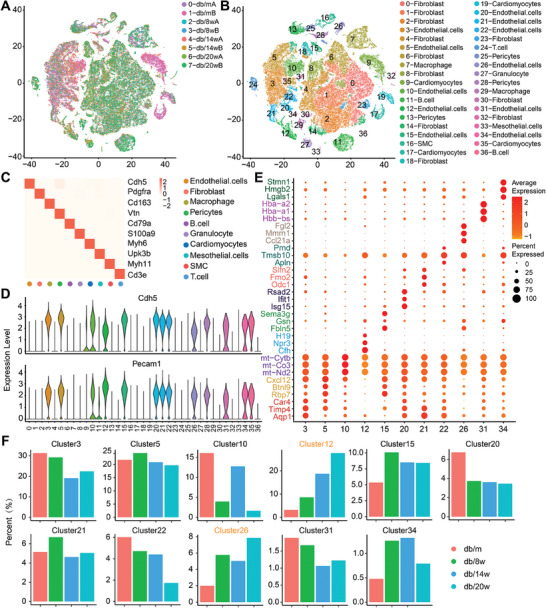
Characterization of cardiac vascular endothelial cells heterogeneity. A) t‐distributed Stochastic Neighbor Embedding (t‐SNE) depicting 63825 single cells isolated from different stages of diabetic heart. Each point represents a single cell, the point color was determined according to the group design. B) t‐SNE projection exhibiting the single cells according to identified cell types. C) Correlation between cell type marker genes and identified cell types. D) Expression of the endothelial cell markers Cdh5 and Pecam1 in different clusters. E) Heat map summarizing the top 3 differentially expressed genes in each cluster of endothelial cells. F) The proportion of various clusters across different pathological stages.

We systematically examined the pathological profiles of diabetic heart injury by sequencing 63825 cells at different stages of diabetic heart injury (**Figure** **1**A,B). Each stage of diabetic heart injury utilized heart samples collected from two distinct mice. Cells were clustered based on their unique molecular markers^[^ ^20^, ^21^, ^22^ ^]^ (Figure 1C,D). In addition to ECs, a significant number of non‐muscle cells were identified, including fibroblasts, pericytes, SMCs, and immune cells, as well as a limited number of cardiomyocytes (which were too large to pass through the single‐cell platform). Using the markers Cdh5 and Pecam1, 16714 cells were designated as ECs and organized into 11 clusters with respective characteristic genes outlined in Figure 1E and Figure S2A (Supporting Information). Cardiac endothelial cells encompass arterial, venous, capillary, lymphatic, and endocardial cells. Based on the pertinent marker genes (arterial: Fbln5; venous: Nr2f2; Capillary: Btnl9; endocardial: Plvap; lymphatic: Lyve1),^[^ ^20^, ^23^ ^]^ we classified the subsets of endothelial cells. Clusters 3, 5, 10, 20, 22, 31, and 34 were predominantly composed of capillary endothelial cells, cluster 21 was derived from veins, while clusters 12, 15, and 26 consisted of endothelial cells of diverse vascular types, without distinct vascular specificity (Figure S2B, Supporting Information).

Based on the cluster identification results, our first analysis focused on the proportion of different clusters in various groups (Figure 1F). Clusters 3, 5, and 10 exhibited the highest percentage of ECs during the early stages of diabetes, constituting over 50% of all ECs. Gene ontology (GO) analysis revealed that these clusters are primarily enriched in fundamental physiological processes of ECs, encompassing angiogenesis, regulation of vessel diameter, and regulation of NO production (Figure S3A–C, Supporting Information). However, cluster 12 accounted for the largest proportion in late‐stage diabetes, comprising 27% of all ECs. Most EC clusters showed a decrease or negligible change in percentage as diabetes progressed, while clusters 12 and 26 exhibited an increasing trend throughout diabetic progression. Based on the results of GO analysis, clusters 12 and 26 are primarily enriched in processes related to inflammation, such as antigen processing and presentation, leukocyte adhesion to endothelial cells, and macrophage activation (Figure S3D,E, Supporting Information). The loss of ECs and infiltration of inflammatory cells play crucial roles in the pathogenesis of vascular injury in diabetes.^[^ ^7^ ^]^ Changes in the proportion of these cellular clusters collectively contribute to the onset and progression of cardiac vascular endothelial injury in diabetes. Furthermore, cluster 20 exhibited functional annotations related to the biological aging process, while cluster 21 was extensively linked with the regulation of oxygen deprivation (Figure S3F–K, Supporting Information).

### 2.2 State Transition of Cardiac Vascular Endothelial Cells During the Progression of Diabetes‐Induced Injury

During the pathological process, cells are exposed to multiple stimuli and undergo various changes in their functional state.^[^ ^24^, ^25^ ^]^ This results in differential gene expression among cells at different states. As cells transit among these states, they undergo transcriptional reprogramming, leading to the silencing of certain genes and the activation of others. To discern the differentiation status of various cell clusters, pseudo time analysis was performed to identify branch points and depict changes in cell processes.

**Figure 2:**
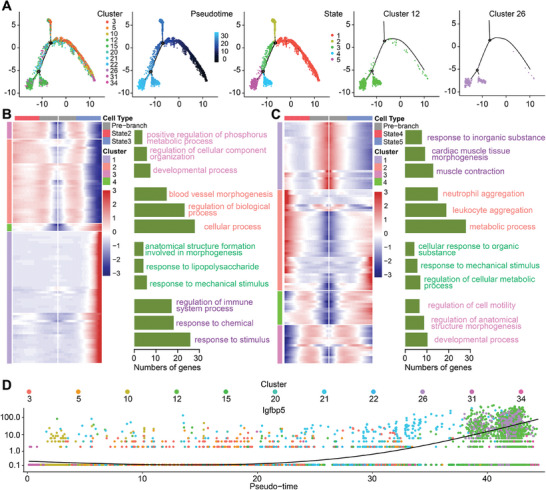
The state transition of cardiac vascular endothelial cells throughout the progression of diabetes‐induced injury. A) Monocle analyses showing the ordering of endothelial cells along pseudo time trajectories. The minute dots in the illustration signify cells, with diverse colors denoting distinct clusters or states. B) The heatmap displaying different blocks of differentially expressed genes along the pseudo time trajectories of the first branch. In terms of cell type, Gray denotes the pre‐brance state, which is state1, Red signifies state2, and Blue indicates state3. The diverse color GO function annotations on the right correspond to the gene set of the corresponding color. C) The heatmap presenting distinct clusters of genes that are differentially expressed in the second branch of the pseudotime trajectory. In terms of cell type, Gray denotes the pre‐brance state, which is state3, Red signifies state4, and Blue indicates state5. The diverse color GO function annotations on the right correspond to the gene set of the corresponding color. D) Changes in Igfbp5 gene expression throughout the pseudo time course, from initiation to termination.

According to the degree of differentiation, all clusters were categorized into five states (**Figure** **2**A). The states for each cluster are detailed in Figure S3L (Supporting Information). It was revealed that clusters 3, 5, 10, 20, 22, and 31 are primarily involved in states 1 and 2, the initial two states involved in cell differentiation. State 3 is mainly characterized by clusters 15, 21, and 34. However, these clusters are not restricted to state 3, as they are also involved in all 5 states. Conversely, clusters 12 and 26 demonstrate a specific association with the latter two states of cell differentiation, specifically states 4 and 5.

At the initial branch point for differentiation, genes regulating the transition from state 1 to state 2 were primarily enriched in biological and cellular processes, with a specific emphasis on blood vessel morphogenesis, according to GO analysis. Meanwhile, the genes regulating the transition from state 1 to state 3 mainly enhance responsiveness to stimuli (Figure 2B). At the second branching site, genes governing the transition from state 3 to states 4 and 5 primarily promote leukocyte infiltration and neutrophil aggregation (Figure 2C). In conjunction with earlier analyses of cellular trajectories, clusters 12 and 26 predominantly represented states 4 and 5. The identification of differential genes at the branching point highlighted crucial factors responsible for the pro‐inflammatory characteristics displayed by clusters 12 and 26. Notably, insulin‐like growth factor binding protein 5 (Igfbp5) is involved in the regulation of differentiation from state 3 to both state 4 and state 5. Igfbp5 is among the top 10 characteristic genes both of cluster 12 and 26 (Figure 2D; Figure S4A,B, Supporting Information).

When it comes to cellular communication among endothelial cell clusters, two modes of interaction were identified (Figure S4C,D, Supporting Information). While all clusters, except for clusters 12 and 26, fell under the first mode, focused signals were primarily revolved around cellular growth and development, such as vascular endothelial growth factor. In contrary, clusters 12 and 26 were associated with the second modality, concentrating mainly on leuco‐endothelial cell adhesion, such as vascular cell adhesion molecules. This further exemplifies the distinct roles of these two cell types across various physiological and pathological processes.

### 2.3 Essential Genes Involved in Cardiac Vascular Endothelial Injury in Diabetes

**Figure 3:**
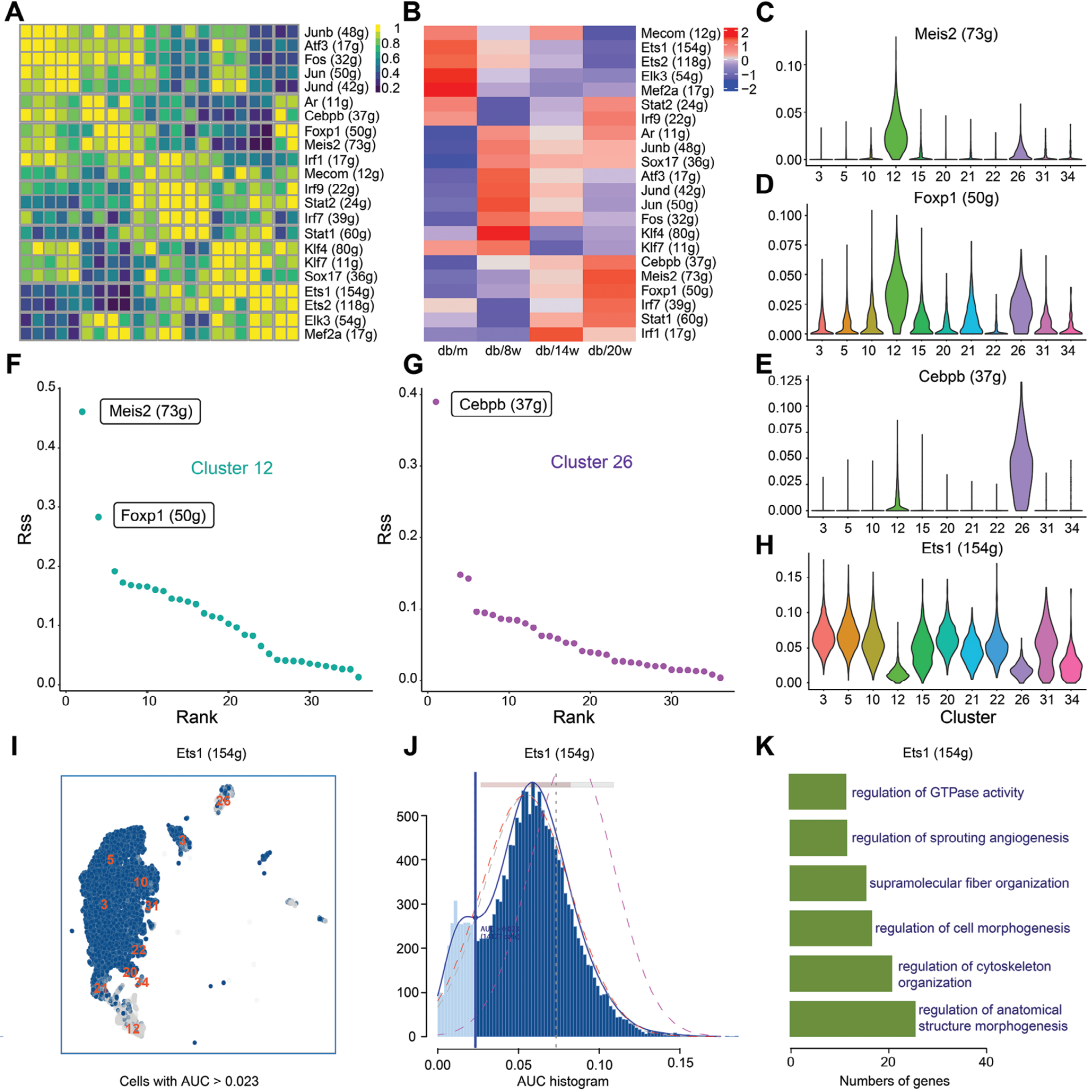
Ets1 is a significant transcription factor that is closely related to angiogenesis. A) SCENIC analysis was employed to reconstruct gene regulatory networks and identify transcription factors. B) Heat map of identified transcription factors by group. C) Expression of the transcription factor Meis2 in different clusters. D) Expression of the transcription factor Foxp1 in different clusters. E) Expression of the transcription factor Cebpb in different clusters. F) Regulatory intensity of cluster 12 transcription factors. G) Regulatory intensity of cluster 26 transcription factors. H–I) Expression of the transcription factor Ets1 in different clusters. J) AUC values of the Regulon, binary expression data representing active status, and expression levels of Ets1 were plotted on t‐SNE coordinates. K) GO analysis of 154 genes regulated by Ets1.

In pursuit of vital genes implicated in cardiac vascular endothelial damage in diabetes, we initially discerned 20 differentially expressed genes within endothelial cells, revealing a progressive augmentation in response to diabetes (Figure S5A, Supporting Information). These genes comprise several characteristic genes of cluster 12, such as complement factor H (Cfh), natriuretic peptide receptor 3 (Npr3), Igfbp5, and cathepsin H (Ctsh). Next, we went on to decipher transcription factors through single‐cell regulatory network inference and clustering (SCENIC), which uncovered a total of 22 transcription factors governing the regulation of endothelial cell differentiation in the face of diabetic injury (**Figure** **3**A). Of particular alert, several transcription factors were dynamically altered with the progression of diabetes, including Meis homeobox 2 (Meis2), Forkhead box P1 (Foxp1), and CCAAT enhancer binding protein beta (Cebpb) (Figure 3B). Interestingly, all these transcription factors were overtly overexpressed in clusters 12 and 26 (Figure 3C–E). Meis2 and Foxp1 were involved in the regulation of 73 and 50 target genes, respectively, and were the top two most significant transcription factors involved in the regulation of cluster 12 (Figure 3F). Cebpb dealt with the regulation of 37 target genes and represented the most significant transcription factor in the regulation of cluster 26 (Figure 3G). Earlier pseudo‐time analysis indicated that clusters 12 and 26 represent the ultimate stages of EC differentiation, and their proportion gradually rose with the progression of the diabetic pathological state. The persistent elevation in expression levels of these three transcription factors may represents the key event underlying this phenomenon.

Furthermore, we noted that ETS proto‐oncogene 1 (Ets1) serves as the transcription factor regulating the largest number of target genes, totaling to 154. Contrary to Meis2, Foxp1, and Cebpb, the expression of Ets1 in endothelial cells gradually declined with the progression of diabetes (Figure 3B), with overtly lower levels in clusters 12 and 26 compared with other clusters (Figure 3H,I). GO analysis indicated that the 154 genes regulated by Ets1 were primarily associated with angiogenesis (Figure 3J,K). The gradual decrease in Ets1 expression may be the primary cause of the reduction in blood vessel density in diabetes. Meis2, Foxp1, and Cebpb are primarily involved in the regulation of clusters 12 and 26, which are closely associated with the inflammatory infiltration of blood vessels. These distinct expression patterns of the two types of transcription factors in endothelial cells work in concert to exacerbate diabetic cardiovascular damage. In this context, rescuing Ets1 may serve as a critical measure to mitigate diabetic cardiovascular injury.

To ascertain whether these vital genes exhibit identical expression changes in diabetes models, excluding the db/db mice, we also established streptozotocin (STZ) and high‐fat diet (HFD) induced diabetes models separately. Body mass and blood glucose levels of experimental animals are presented in Tables S3 and S4 (Supporting Information). We evaluated modifications in the expression of Cfh, the most significant characteristic gene of cluster 12, and Ets1, the most active transcription factor, in these models. Our results indicated that alterations in the expression of Cfh and Ets1 in the cardiovascular endothelium from both diabetic models were consistent with the manifestations observed in the sequencing data (Figure S5B–M, Supporting Information). This further corroborated the reliability of our experimental findings.

### 2.4 Ets1 Attenuates Cardiac Vascular Endothelial Injury in db/db Mice

To examine the role of Ets1 in diabetes‐associated endothelial injury, mouse Ets1 overexpressing adeno‐associated virus‐9 genome particles containing the TIE promoter (AAV9‐m‐TIE‐Ets1‐Flag‐EGFP, abbreviated to AAV9‐Ets1) and control virus (AAV9‐NC) were injected via the tail vein into 8‐week‐old db/db or db/m mice. At 20 weeks of age, Flag and CD31 staining of heart tissues revealed > 90% AAV9 transfection efficiency in cardiac endothelial cells (Figure S6A, Supporting Information). Furthermore, the fluorescence intensity of Ets1 in CD31‐positive domains of cardiac tissues treated with AAV9‐Ets1 was noticeably augmented by a substantial factor of 3.2 compared to the AAV9‐NC group (Figure S6B,C, Supporting Information). These findings consolidate the effective overexpression of Ets1 using AAV9‐Ets1 in cardiac vascular endothelial cells.

**Figure 4:**
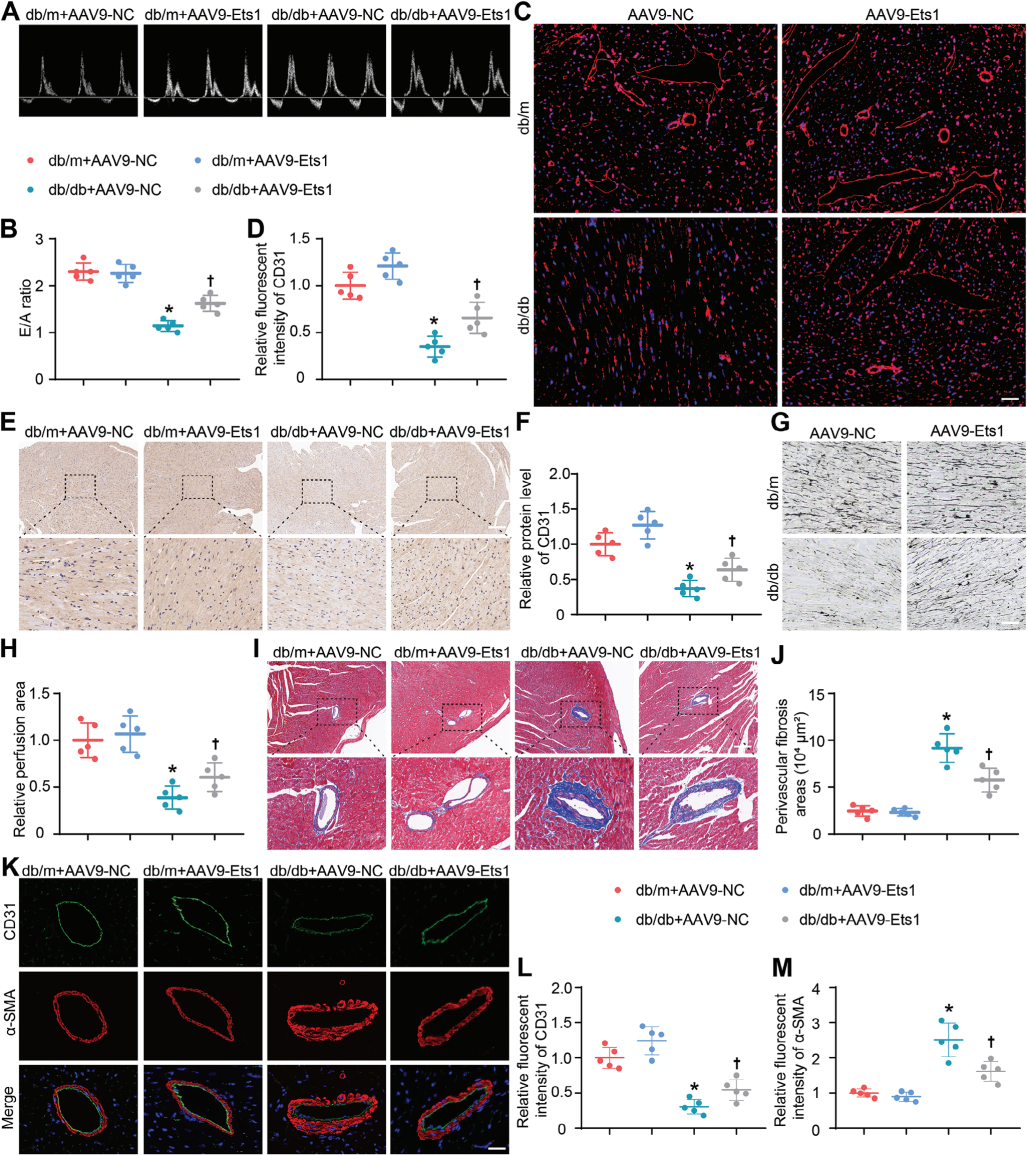
Endothelial Ets1 rescue restores cardiac vascular density in db/db mice. A,B) Representative Doppler echocardiography images and quantification of the ratio between the early and late mitral diastolic waves (E/A ratio) (n = 5). C,D) Representative and quantified immunofluorescence staining of CD31 (n = 5). The scale bars depict a length of 50 µm. E,F) Representative and quantified immunohistochemical staining of CD31 (n = 5). The scale bars depict a length of 250 µm. G) Representative vascular image detection using ink staining. The scale bars depict a length of 100 µm. H) Quantification of ink perfusion area (n = 5). I–J) Representative and quantified Masson's trichrome staining of perivascular tissue (n = 5). The scale bars depict a length of 300 µm. K–M) Representative and quantified immunofluorescence staining of CD31 and α‐SMA (n = 5). The scale bars depict a length of 30 µm. ^*^ *P* < 0.05 versus db/m+AAV‐NC; ^†^ *P* < 0.05 versus db/db+AAV‐NC.

Subsequent studies confirmed an apparent deterioration of diastolic cardiac function as evaluated by ultrasound examination among the db/db mice compared to the db/m mice. Significantly, administration of AAV9‐Ets1 remarkably ameliorated diastolic function in db/db mice when compared to AAV9‐NC (**Figure** **4**A,B). Given that diastolic dysfunction occurred preceding systolic dysfunction in diabetic mice, no alteration in systolic function was observed in our diabetic models during this time frame (Figure S6D,E, Supporting Information). Numerous studies have highlighted the critical role of Ets1 in angiogenesis.^[^ ^26^, ^27^ ^]^ Our immunohistochemical and immunofluorescence results also indicated that AAV9‐Ets1 upregulated CD31 levels in cardiac tissues (Figure 4C–F). Furthermore, our ink staining data exhibited that endothelial Ets1 overexpression significantly reversed the reduced cardiac vascular density in db/db mice (Figure 4G,H). In addition, the efficacy of Ets1 overexpression in ameliorating perivascular fibrosis in db/db mice was verified using Masson trichrome staining (Figure 4I,J) and immunofluorescence staining (Figure 4K–M). The decreased expression of endothelial marker (CD31) and elevated expression of mesenchymal cell marker (α‐SMA) indicate that Ets1 suppressed microvascular endothelial‐mesenchymal transition (EndMT) in diabetic heart.

**Figure 5:**
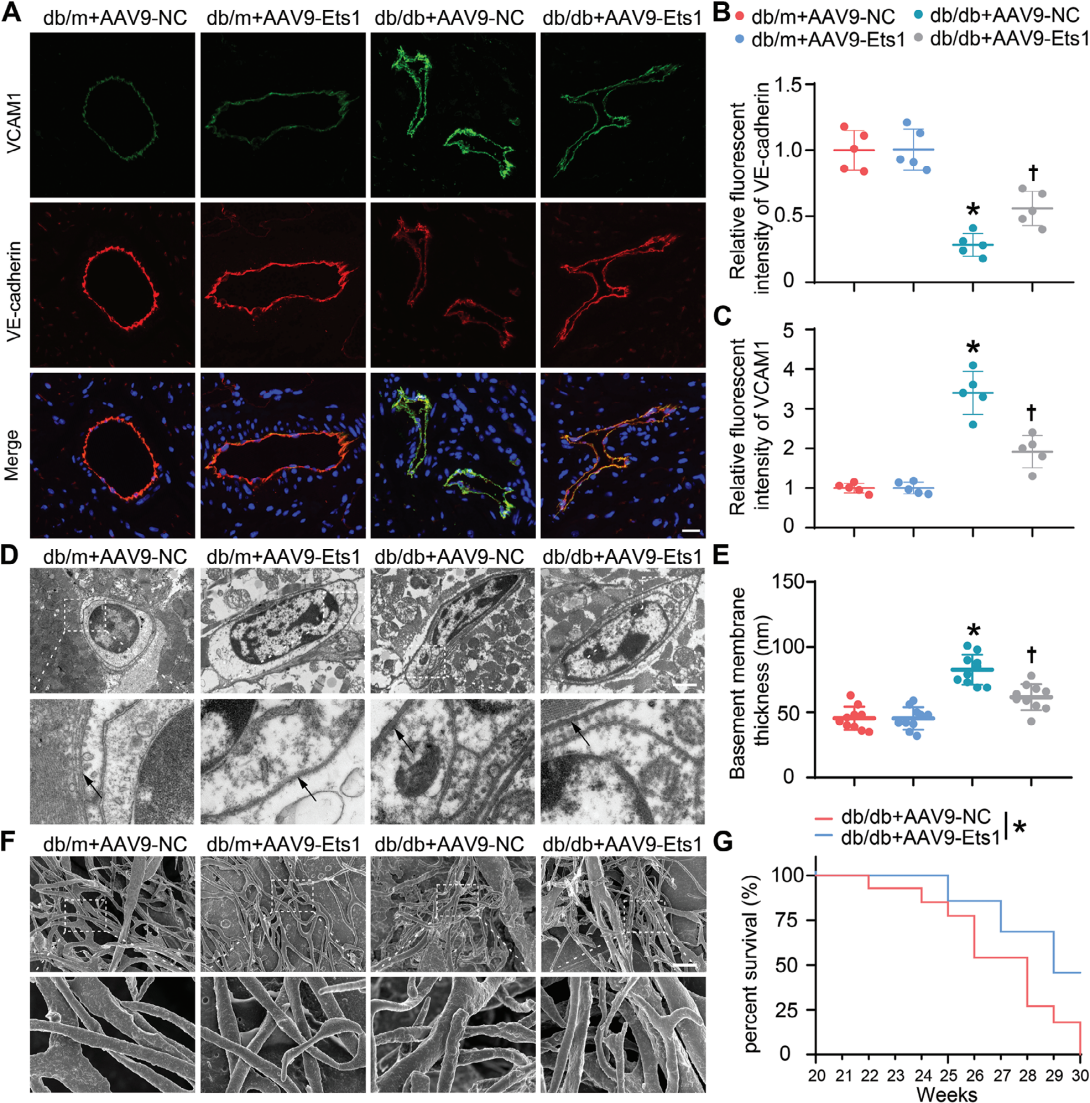
Endothelial Ets1 rescue attenuates cardiac vascular endothelial injury in db/db mice. A) Co‐immunofluorescence staining of VE‐cadherin and VCAM1 was conducted to assess endothelial damage (n = 5). Scale bars = 20 µm. B,C) Quantification of the expression level of VE‐cadherin and VCAM1. D) Transmission electron microscope was performed to observe changes in the endothelial cells. Scale bars = 2 µm. E) Quantification of the basement membrane thickness (n = 10). ^*^ *P* < 0.05 versus db/m+AAV‐NC; ^†^ *P* < 0.05 versus db/db+AAV‐NC. F) Representative scanning electron micrographs of cardiac vascular corrosion in different groups. Scale bars = 10 µm. G) Survival curves of the animals (n = 10). *P < 0.05 versus db/db+AAV‐NC.

VE‐cadherin is crucial for maintaining vascular endothelium integrity, essential for establishing close junctions, gap junctions, adhesive junctions, and ligamentous junctions among endothelial cells.^[^ ^28^ ^]^ Immunofluorescence analysis revealed a substantial decrease in VE‐cadherin expression in myocardial vascular endothelium of db/db mice, which was successfully reversed by Ets1 overexpression (**Figure** **5**A,B). Furthermore, the increased expression of Ets1 significantly slowed down the progression of vascular cell adhesion molecule1 (VCAM1), a molecule instrumental in inducing leukocyte adhesion to the endothelium and responsible for the thickening of the basement membrane (Figure 5C). In addition to this, the inflammatory factors in the cardiac tissue of db/db mice were also alleviated by Ets1 (Figure S6F–H, Supporting Information). Further analysis using transmission electron microscopy showed that Ets1 overexpression significantly reduced the thickness of the basement membrane in cardiac vascular endothelial cells from db/db mice (Figure 5D,E). Additionally, a scanning electron microscope was used to visualize the vascular corrosion casts. Our findings indicated that overexpression of Ets1 significantly improved the vascular morphology in db/db mice, resulting in a reduction of bumps and dips in the vascular surface (Figure 5F). These observations strongly support a protective role for Ets1 on endothelial cells in diabetes‐related cardiovascular injury. Given the beneficial effect of Ets1 on vasculature, it is not surprising that elevated Ets1 expression in endothelial cells was linked to a significantly improved survival rate in db/db mice (Figure 5G).

### 2.5 Ets1 Overexpression Alleviates Kidney Injury in db/db Mice

**Figure 6:**
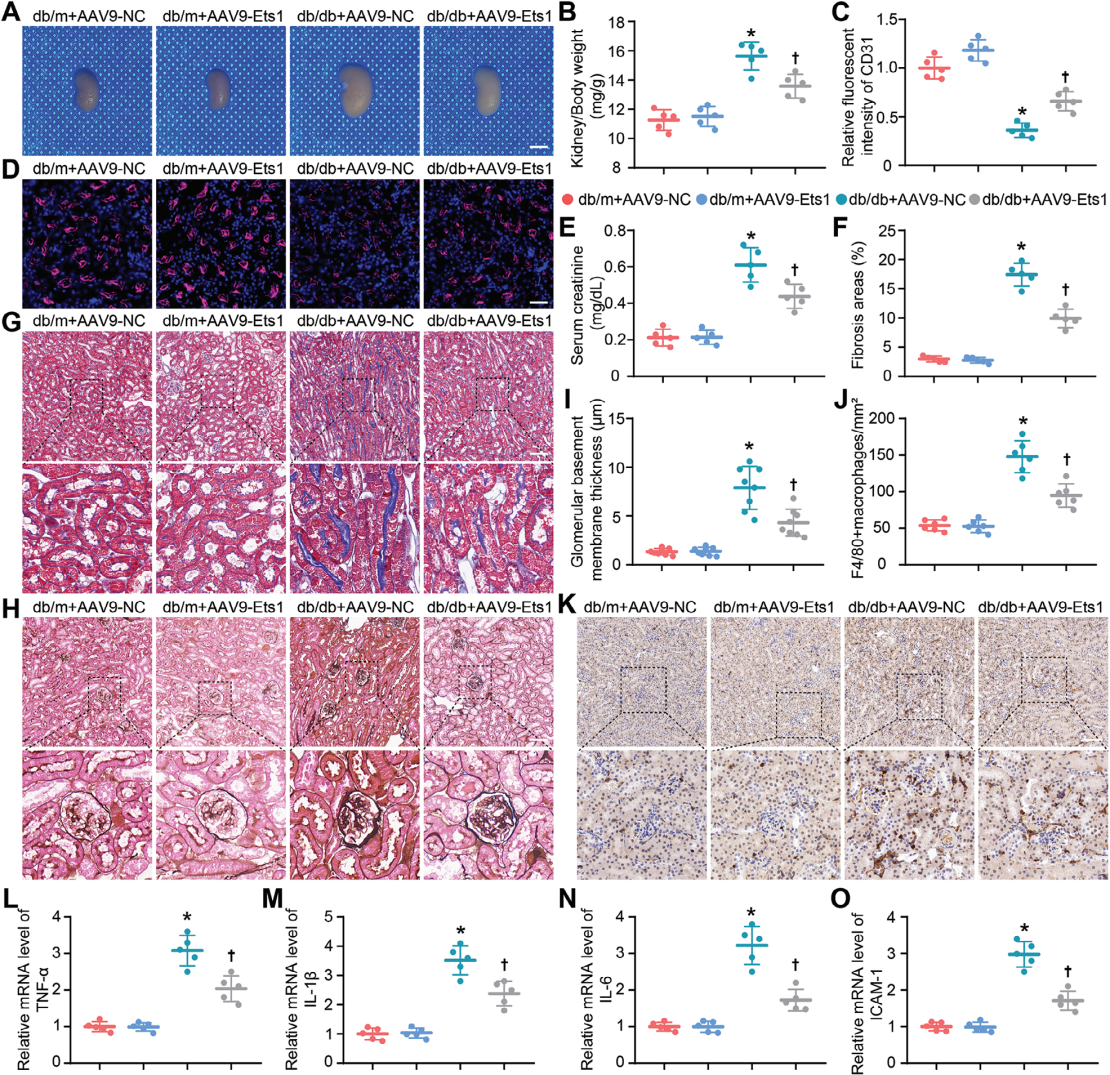
Ets1 overexpression in endothelial cells alleviates renal injury in db/db mice. A) Representative kidney images from different groups. B) Quantification of kidney/body weight (n = 5). Scale bars = 400 mm. C,D) Representative and quantified immunohistochemical staining of CD31 (n = 5). Scale bars = 100 µm. E) Serum creatinine in different groups (n = 5). F,G) Representative and quantified Masson's trichrome staining of kidneys (n = 5). Scale bars = 100 µm. H) Representative periodic acid‐silver methenamine staining of kidneys (n = 8). Scale bars = 100 µm. I) Quantification of glomerular basement membrane thickness. J–K) Representative and quantified F4/80 staining of kidneys (n = 6). Scale bars = 100 µm. L–O) The relative mRNA level of TNF‐α, IL‐1β, IL‐6, and ICAM‐1 in kidneys (n = 5). ^*^ *P* < 0.05 versus db/m+AAV‐NC; ^†^ *P* < 0.05 versus db/db+AAV‐NC.

During the dissection of mice under anesthesia, an unforeseen phenomenon was uncovered: administration of AAV‐Ets1 provoked a marked reduction in renal hypertrophy in db/db mice (**Figure** **6**A,B). Initially, co‐staining of Flag and CD31 validated the successful transfection of AAV9‐Ets1 in kidneys (Figure S6I, Supporting Information), and the apparent upregulation of Ets1 expression increased CD31 levels in the kidneys of db/db mice (Figure 6C,D). Critically, AAV9‐Ets1 significantly decreased serum creatinine levels in db/db mice, compared to the AAV9‐NC group (Figure 6E). Data from Masson trichrome staining indicated that AAV9‐Ets1 noticeably reduced renal fibrosis in db/db mice compared to the AAV9‐NC group (Figure 6F,G). Overexpression of Ets1 in endothelial cells also diminished the thickening of the glomerular basement membrane in db/db mice, as evaluated using periodic acid‐silver methenamine staining (Figure 6H,I). Moreover, the intervention of AAV9‐Ets1 resulted in notable reductions in macrophage infiltration (Figure 6J,K) and inflammatory factors (Figure 6L–O), critical pathogenic elements in diabetic nephropathy. These findings indicate that endothelial Ets1 overexpression protects against diabetic renal damage. Therefore, the improved survival in db/db mice following AAV9‐Ets1 treatment may also be attributed to the beneficial outcomes in the kidney.

### 2.6 Ets1 Knockdown Exacerbates Cardiac and Renal Injury in db/db Mice

**Figure 7:**
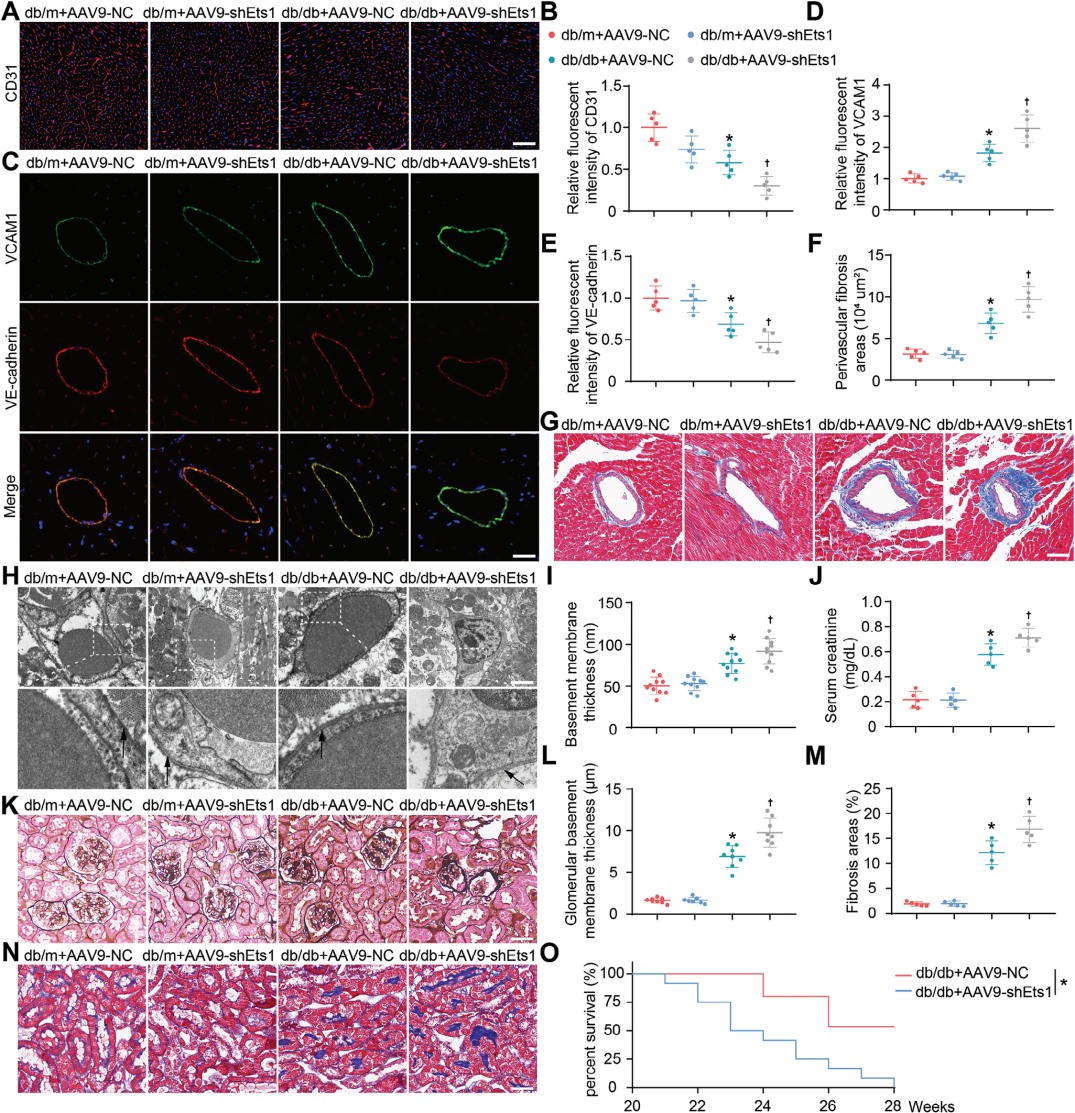
Ets1 knockdown in endothelial cells exacerbates cardiac and renal injury in db/db mice. A,B) Representative and quantified immunofluorescence staining of CD31 in the heart (n = 5). The scale bars depict a length of 100 µm. C) Co‐immunofluorescence staining of VE‐cadherin and VCAM1 in the heart was conducted to assess endothelial damage (n = 5). Scale bars = 20 µm. D,E) Quantification of the expression level of VE‐cadherin and VCAM1. F,G) Representative and quantified Masson's trichrome staining of perivascular tissue in the heart (n = 5). The scale bars depict a length of 50 µm. H) Transmission electron microscope was performed to observe changes in cardiac endothelial cells. Scale bars = 2 µm. I) Quantification of the basement membrane thickness (n = 10). J) Serum creatinine in different groups (n = 5). K) Representative periodic acid‐silver methenamine staining of kidneys (n = 8). Scale bars = 60 µm. L) Quantification of glomerular basement membrane thickness. M,N) Representative and quantified Masson's trichrome staining of kidneys (n = 5). Scale bars = 60 µm. *P < 0.05 versus db/m+AAV‐NC; ^†^ *P* < 0.05 versus db/db+AAV‐NC. O) Survival curves of the animals (n = 10). ^*^ *P* < 0.05 versus db/db+AAV‐NC.

To reinforce the significance of Ets1 in diabetic cardio‐renal injury, we suppressed Ets1 expression in the endothelium of db/db mice in vivo using AAV9‐shEts1. Following 12 weeks of caudal vein injection of AAV9 in 8‐week‐old mice, the overlap rate of CD31 and Flag in heart and kidney tissues exceeded 90%, indicating that AAV9 had high endothelial specificity and was not transfected into other cell types (Figure S7A,B, Supporting Information). Subsequent work demonstrated that, compared to AAV9‐NC, AAV9‐shEts1 reduced Ets1 expression within the vascular endothelium by ≈70% on average (Figure S7C,D, Supporting Information). We then evaluated the effect of AAV9‐shEts1 on cardiac vascular density using CD31 staining and found that AAV9‐shEts1 significantly reduced the vascular density of db/db mice, further confirming an important role of Ets1 in angiogenesis (**Figure** **7**A,B). Meanwhile, AAV9‐shEts1 decreased the expression of VE‐cadherin and increased the expression of VCAM1 in the vascular endothelium of db/db mice, suggesting that AAV9‐shEts1 exacerbated vascular endothelium injury of db/db mice (Figure 7C–E). Moreover, Masson trichrome staining and immunofluorescence staining indicated that AAV9‐shEts1 exacerbated perivascular fibrosis in db/db mice, compared to AAV9‐NC treatment (Figure 7F,G; Figure S7E–G, Supporting Information). At the microscopic level, AAV9‐shEts1 increased the basement membrane thickness of vascular endothelium in db/db mice and led to more irregular vascular morphology in db/db mice (Figure 7H,I; Figure S8A, Supporting Information). Given the severe damage inflicted upon blood vessels, AAV9‐shEts1 also further deteriorated cardiac function in db/db mice (Figure S8B,C, Supporting Information).

The experimental outcomes of AAV9‐shEts1 in the kidney have further substantiated the role of ETS1 in vascular injury. Initially, it was observed that AAV9‐shEts1 significantly increased kidney‐to‐body weight ratio in db/db mice, while lab biomarkers indicated that AAV9‐shEts1 elevated serum creatinine levels in db/db mice (Figure 7J; Figure S8D,E, Supporting Information). Subsequently, the influence of AAV9‐shEts1 on renal vascular density was evaluated through CD31 staining. In line with our earlier findings, AAV9‐shEts1 remarkably reduced CD31 expression in the kidneys of db/db mice (Figure S8F–G, Supporting Information). The impairment evoked by AAV9‐shEts1 in renal blood vessels also resulted in the thickening of the glomerular basement membrane and increased renal fibrosis (Figure 7K–N). Notably, AAV9‐shEts1 reduced survival in db/db mice (Figure 7O). These findings suggest that diminished Ets1 expression exacerbates cardiac and renal damage in db/db mice.

## 3 Discussion

Cardiovascular disease is the leading cause of diabetes‐associated mortality.^[^ ^5^, ^6^ ^]^ Cardiac vascular endothelial cells, which are the primary cellular component affected by diabetic injury, respond to the demands of the surrounding microenvironment, leading to the emergence of different endothelial cell types.^[^ ^14^ ^]^ In this study, a single‐cell transcription landscape of cardiac vascular endothelial cells in db/db mice was mapped. Two pivotal cell clusters were identified, along with their crucial regulatory factors in response to diabetic injury. Potential biomarkers of cardiac vascular endothelial injury in db/db mice were also unveiled. Moreover, evidence was provided demonstrating the protective role of the important transcription factor Ets1 in mitigating vascular endothelial injury in db/db mice.

The heterogeneity of human and mouse cardiac endothelial cells has garnered substantial attention, yet most emphasis has been on normal or developing hearts.^[^ ^20^, ^22^, ^29^ ^]^ Li and colleagues employed single‐cell sequencing to evaluate intercellular communication between cardiac cells in diabetic mice induced by STZ, however, this work predominantly focused on fibroblasts, and the heterogeneity of endothelial cell populations was not precisely delineated.^[^ ^30^ ^]^ McCracken and associates identified 11 clusters of endothelial cells within the developing heart and mapped these clusters to one or multiple vascular subtypes (arterial, venous, capillary, lymphatic, and endocardial).^[^ ^31^ ^]^ However, these clusters lacked significant pro‐inflammatory activity and were predominantly linked to the progression of blood vessels. This aligns with our findings in db/m mice, where diabetes might stimulate the emergence of inflammatory endothelial cell subsets. In the context of transcription factor analysis, our study also revealed some uniformity. For example, Mecom was identified as the most abundant transcription factor within arterial endothelial cells. Nonetheless, the issue of cardiac endothelial cell heterogeneity in diseases such as hypertension and myocardial infarction requires substantial deliberation in the forthcoming years.

In our study, we identified 11 combined clusters of endothelial cells among four groups of mice. These clusters were categorized into two main domains based on their developmental patterns, communication mechanisms, and gene expression profiles. The first group consists of clusters 3, 5, 10, 15, 20, 21, 22, 31, and 34, which are primarily involved in biological processes of angiogenesis, regulation of vascular diameter, and maintenance of endothelial barrier function. The second group includes clusters 12 and 26, which primarily facilitate the adhesion of immune cells to endothelial cells. As diabetes progresses, the proportion of the second type of cells increases, while the first type of cells decreases. By analyzing cluster signatures and transcription factors, we were able to identify key targets for the treatment and assessment of diabetic cardiac endothelial injury.

Based on our results, Ets1 is the most prominent transcription factor involved in the regulation of target genes and is highly expressed in clusters 3, 5, 10, 15, 20, 21, 22, 31, and 34, while it is poorly expressed in clusters 12 and 26. The Ets1 transcription factor belongs to the Ets gene family and is recognized for its regulatory function on vascular development.^[^ ^32^ ^]^ Recent research has highlighted the importance of endothelial Ets1 in the development of coronary vessels.^[^ ^26^ ^]^ Studies conducted by Wang and the team have revealed that loss of Ets1 reduces the expression of multiple genes related to angiogenesis, leading to compromised coronary vessel development. In addition to this, Ets1 promotes endothelial barrier function in response to inflammatory stimuli by enhancing the expression of VE‐cadherin.^[^ ^33^ ^]^ In the central nervous system, endothelial Ets1 is recognized as an essential regulator of endothelial‐mesenchymal transition. Down‐regulation of Ets1 triggers EndMT and exacerbates blood‐brain barrier dysfunction in multiple sclerosis.^[^ ^34^ ^]^ Furthermore, numerous investigations corroborate the crucial involvement of Ets1 in orchestrating EndMT progression.^[^ ^35^, ^36^ ^]^ Nevertheless, there appears to be disparate roles for Ets1 in EndMT progression as reported by these studies, potentially due to variations in structural differences, microenvironmental conditions, and experimental design. In the previous study of EndMT associated with diabetes mellitus,^[^ ^36^ ^]^ the focus was predominantly on human umbilical vein endothelial cells (HUVECs) and the level of Ets1 expression in HUVECs increased in response to high glucose stimulation. However, this research did not incorporate in vivo experimentation. Our present results indicate that cardiac vascular endothelial Ets1 is down‐regulated during the course of diabetes progression, and overexpression of Ets1 in endothelial cells can alleviate diabetic vascular sparsity, fibrosis, inflammatory infiltration, and overall vascular dysfunction. Taken together, these studies underscore the vital role of Ets1 in endothelial cells during pathophysiological processes.

Findings from our study identified clusters 12 and 26 as the final state of cardiac endothelial differentiation in diabetes. Several characteristic genes from these two clusters exhibited high expression profiles during diabetes development. For instance, Plvap is the characteristic gene of cluster 12 and a protein unique to endothelial cells.^[^ ^37^, ^38^ ^]^ Earlier findings have indicated that Plvap is the sole protein component responsible for the formation of diaphragm in endothelial microdomains and plays a crucial role in transcellular migration.^[^ ^39^, ^40^ ^]^ Furthermore, Plvap facilitates the passage of leukocytes through the endothelial layer to reach sites of inflammation.^[^ ^41^ ^]^ This phenomenon could explain why Plvap is deemed the hallmark gene of cluster 12.

Intriguingly, findings from our work ranked Igfbp5, a secretory protein widely reported in cell adhesion and tissue fibrosis,^[^ ^42^ ^]^ among the top 10 characteristic genes of both 12 and 26 clusters. In diabetic studies, Igfbp5 has been shown to aggravate diabetic kidney injury by activation of proinflammatory responses in glomerular endothelial cells.^[^ ^43^ ^]^ Additionally, increased expression of Igfbp5 plays a key role in the development of diabetic neuropathy.^[^ ^44^ ^]^ Work from the heart has noted Igfbp5 as a key gene in both dilated cardiomyopathy and hypertrophic cardiomyopathy based on microarray data.^[^ ^45^ ^]^ Furthermore, Igfbp5 has been shown to mediate high glucose‐induced activation of cardiac fibroblasts, promoting cardiac fibrosis.^[^ ^46^ ^]^ However, the role of Igfbp5 in cardiac vascular endothelial cells has not been reported. Our data demonstrate that Igfbp5 expression levels in cardiac vascular endothelial cells are consistently elevated throughout the course of diabetes progression, indicating that Igfbp5 is an important target role for the treatment of diabetic injury in both hearts and kidneys. Further study is needed to elucidate the mechanism behind Igfbp5‐triggered regulation of organ function.

Currently, the clinical evaluation of diabetic cardiac vascular function mainly relies on invasive methods such as the assessment of blood flow velocity reserve and microcirculation resistance index.^[^ ^47^ ^]^ Unfortunately, there is no definitive blood biomarker available to accurately evaluate vascular damage in the heart. Cfh is a serum glycoprotein and a major regulator of the complement system alternative pathway, playing a pivotal role in maintaining immune homeostasis.^[^ ^48^ ^]^ Recent studies by Wang and coworkers examined biomarkers of type 2 diabetes (T2D) events through 2 nested case‐control studies and circulating proteomics. They identified seven proteins, including Shbg, Cand1, Apof, Sell, Mia3, Ighv1‐2, and Cfh, as proteomic biomarkers of T2D events.^[^ ^49^ ^]^ Another study exploring proteomics in peripheral blood and diabetic mellitus type 2 (T2DM) risk incidents also recognized Cfh as a cardinal indicator of T2DM risk. By analyzing initial data from 2839 samples from the Framingham Heart Study (FHS) and Malmo Diet and Cancer Study (MDCS), the analysis pinpointed 19 proteins correlated with T2DM risk, including Cfh, after adjusting for clinical risk factors twice.^[^ ^50^ ^]^ Additionally, Brahimaj and colleagues evaluated the correlation between 26 plasma inflammatory markers and the clinical progression of diabetes in 971 patients, revealing that five proteins, including Cfh, were notably associated with the early progression of diabetes.^[^ ^51^ ^]^ In our current study, Cfh emerged as the most distinctively differentially expressed gene among 12 subgroups of endothelial cells, and its expression level continued to increase with progressive diabetes. This suggests that Cfh could serve as a biomarker for vascular damage in diabetes. The correlation between circulating Cfh protein levels in patients with diabetes and cardiac vascular injury requires validation with further clinical trials.

In conclusion, results from our study provide a comprehensive understanding for the onset and development of diabetic cardiac vascular endothelial damage and the heterogeneity of the involved cells. These findings offer valuable insights into potential treatments and assessments of diabetic cardiovascular endothelial damage.

## 4 Experimental Section

### Animal Models

Animal experiments were performed according to the Guide for the Care and Use of Laboratory Animals, eighth edition (2011), and were approved by the Fourth Military Medical University Ethics Committee on Animal Care (FMMU‐20201017). Male db/db and db/m mice used in this study were procured from Gempharmatech Co., Ltd. (China). Mice were housed in the institutional animal facility with a 12‐h light and 12‐h dark circadian rhythm at 22 °C ± 1 °C and 60 ± 5% humidity. Mice were allowed free access to food and water. Blood glucose levels were monitored weekly using a reflectance meter (Accu‐Chek, Germany). The early diastolic wave/late diastolic wave (E/A) proportion and left ventricular ejection fraction (LVEF) were assessed using an echocardiography system (VisualSonics Inc., Canada) to evaluate cardiac function, as previously indicated.^[^ ^52^ ^]^

### Single‐Cell RNA‐Sequencing

Hearts were rinsed with pre‐cooled PBS, minced, and single‐cell suspensions were prepared by dissociating tissues with 0.25% trypsin (Hyclone, USA) and 10 µg/mL recombinant DNase I (RNase‐free) (TaKaRa, Japan). Utilizing the 10x Genomics ChromiumTM platform, Unique Molecular Identifier (UMI) and cell‐barcoded beads were loaded to saturation, ensuring one‐to‐one pairing of each cell with a bead in a Gel Beads‐in‐Emulsion (GEM). Following exposure to a cell lysis buffer, polyadenylated RNA molecules were hybridized to the beads. Beads were retrieved into a single tube for reverse transcription and each cDNA molecule was tagged on the 5′ end with UMI and cell label indicating cell origin. Sequencing libraries were quantified using a High Sensitivity DNA Chip (Agilent, USA) on a Bioanalyzer 2100 and the Qubit High Sensitivity DNA Assay (Thermo Fisher Scientific, USA). Libraries were sequenced on NovaSeq6000 (Illumina, USA), generating 150 bp paired‐end reads. The readout was then processed using the Cell Ranger 4.0 pipeline with default and recommended parameters. For more detailed information, please refer to the supplementary methods.

### Intervention of Ets1 in Endothelial Cells

To generate a mouse model with endothelial cell (EC)‐specific overexpression of Ets1, adeno‐associated virus‐9 genome particles containing the TIE1 promoter, Flag, and EGFP (abbreviated as AAV9‐Ets1) were conducted by Hanbio Technology (Shanghai, China). A total of 100 µL AAV9–Ets1 or AAV9‐NC at a density of 2.0 × 10^12 v.g. ml^−1^ was delivered into 8‐week‐old db/db mice through the caudal vein. Similarly, mouse Ets1 knockdown adeno‐associated virus‐9 genome particles containing the TIE1 promoter, Flag, and EGFP (AAV9‐m‐TIE1‐shEts1‐Flag‐EGFP, abbreviated to AAV9‐shEts1) were used to knock down endothelial Ets1 in mice. The sequences of shRNA for Ets1 are 5′‐ CCGGAGGTGGTAGACTTCCATTCCACTCGAGTGGAATGGAAGTCTACCACCTTTTTTG‐3′. The transfection efficacy of AAV9 in endothelial cells was assayed via the co‐fluorescence of Flag and CD31, Ets1 and CD31.

### Immunohistochemistry and Immunofluorescence Staining

Immunohistochemistry staining of cardiac tissues was performed as previously described.^[^ ^53^ ^]^ In brief, heart sections were incubated with respective primary antibodies at 4 °C overnight, followed by incubation with secondary antibodies at 37 °C for 1 h and detection using 3, 3′‐diaminobenzidine. Finally, the sections were counterstained with hematoxylin prior to microscopic examination.

For immunofluorescence staining, sections were rendered permeable by 0.5% Triton X‐100, and were subsequently blocked with 1% bovine serum albumin (BSA) at 37 °C for 1 h. The sections were then incubated with primary antibodies at 4 °C for 16 h, followed by three washes with PBS (10 min each). Samples were cultivated with secondary antibodies for another hour at 37 °C. After washing with PBS, DAPI working solution was applied to the samples at 37 °C for 10 min. Finally, the antifade solution was added and the images were visualized under a fluorescence microscope (Nikon Eclipse C1, Tokyo, Japan). Antibody information is shown in Table S1 (Supporting Information) (Table S1, Supporting Information).

### Scanning Electron Microscopy

Scanning electron microscopy was employed to assess the integrity of microvascular endothelium in the vessel wall of mouse hearts. A low‐viscosity resin mixed with benzoyl peroxide was perfused into the heart at a pressure of less than 100 mmHg through aorta. Hearts were then submerged in a 5% solution of sodium hydroxide at room temperature, with the solution being replaced every 12 h for 4–5 days. After the removal of connective tissues surrounding the vessels, standard pretreatment procedures including dehydration, desiccation, and gilding were performed prior to visualization of samples through a Hitachi‐S4800 (Tokyo, Japan) scanning electron microscope.

### Transmission Electron Microscopy

The left ventricles of the mouse heart were initially perfused with chilled PBS to remove blood, followed by fixation with 2.5% (wt/vol.) glutaraldehyde in a 0.1 mol L^−1^ phosphate buffer (pH 7.4, 4 °C) for 24 h. Subsequently, heart samples were post‐fixed with 1% (wt/vol.) osmium tetroxide in a 0.1 mol L^−1^ sodium cacodylate buffer (pH 7.4, 4 °C) for 2 h. Samples were then dehydrated, embedded, sectioned, and stained. All images were obtained using a transmission electron microscope (JEOL Ltd., Tokyo, Japan).

### Quantitative Real‐Time PCR (qPCR)

qPCR was performed as described previously.^[^ ^53^ ^]^ Total RNA of ECs isolated from murine models or cultivated within six‐well plates was extracted utilizing TRIzol (Invitrogen), and reversely transcribed into cDNA using the PrimeScriptRT Reagent Kit (TaKaRa, Dalian, China). qPCR was executed using SYBR Green (Bio‐Rad Laboratories, CA, USA). All steps were implemented in compliance with the manufacturer's guidelines. The primer sequences for qPCR are listed in Table S2 (Supporting Information) (Table S2, Supporting Information).

### Microvascular Imaging Using Gelatin‐Ink Perfusion

Perfusion with gelatin ink was conducted by first heating the ink to 37 °C followed by the addition of 1% gelatin. The solution was then perfused via caudal vein. Once the limbs had turned black, the great vessels at the cardiac base, as well as the superior and inferior vena cava, were separated and ligated. Subsequently, hearts were chilled at 4 °C for at least an hour before removal and fixation in 4% paraformaldehyde, followed by cryosectioning.

### Histological Analysis

Mouse cardiac or kidney tissues were fixed overnight with 4% paraformaldehyde, embedded in paraffin, and cut into 8‐µm slices. Masson's trichrome staining was conducted to assess collagen content, as described previously.^[^ ^53^ ^]^ Periodic acid‐silver methenamine (PASM) staining is used for meticulous observation of the thickness of the glomerular basement membrane in the kidney, please refer to the supplementary methods for details.

### Statistical Analysis

Data are expressed as means ± standard deviation of the mean (SD). The Shapiro‐Wilk test was used to assess data normality. An unpaired Student's t‐test was employed to identify the difference between two groups. Differences among multiple groups were evaluated using one‐way ANOVA followed by the Tukey *post‐hoc* test. All statistical analyses were performed using GraphPad Prism 8 (GraphPad Software, San Diego, CA, USA). A P value of less than 0.05 was considered statistically significant.

## Conflict of Interest

The authors declare no conflict of interest.

## Supporting information



Supporting Information

## Data Availability

The data that support the findings of this study are available from the corresponding author upon reasonable request.
