# Supplementary material for: Single‐Cell RNA Sequencing Uncovers Pathological Processes and Crucial Targets for Vascular Endothelial Injury in Diabetic Hearts
Source: Adv Sci (Weinh). 2024 Oct 30;11(47):2405543. doi: 10.1002/advs.202405543 (PMC11653609; doi:10.1002/advs.202405543)
Supplement: Supplementary file 1 — Supporting Information [file ADVS-11-2405543-s001.docx]

**Supplemental Data**

**Single cell RNA-seq data processing**

Raw data were processed using the Cell Ranger 4.0 sequence workflow with default and suggested settings. FASTQ files from Illumina sequencing were aligned to the mouse genome, version GRCm38 using the STAR algorithm. Gene-Barcode arrays were then generated for each sample by counting Unique Molecular Identifiers (UMIs) and excluding non-cellular associated barcodes. This process resulted in gene-barcode arrays encapsulating the encoded cells and gene expression counts. This output was subsequently imported into the Seurat (v3.2.0) R package for quality control and further analysis of our single-cell RNAseq data. All procedures were executed using default parameters, unless specified otherwise. Initially, the matrices were refined to remove low-quality cells based on three quality criteria: (1) the number of detectable transcripts (number of unique molecular identifiers); (2) the number of identified genes; and (3) the ratio of reads mapping to mitochondrial genes, using quartile threshold screening criteria. Mitochondrial gene expression was approximated utilizing the Percentage Feature Set function within the Seurat package. The data were then normalized using the Normalize Data function in Seurat, and a subset of variables genes were isolated. These variable genes were identified while controlling for the robust correlation between variabilities and mean expression. Following this, data from different samples were integrated by identifying ‘anchors’ between datasets using the Find Integration Anchors and Integrate Data functions in Seurat. Principal Component Analysis (PCA) was performed, and the data were reduced to the top 30 PCA components following data scaling. Finally, clusters were visualized in a 2D representation created with t-distributed stochastic neighbor embedding (t-SNE).

**Identification of cell types and subtypes by nonlinear dimensional reduction (t-SNE)**

Cell aggregation was conducted through graph-centric clustering of the PCA-reduced data using the Louvain Algorithm after constructing a shared nearest neighbor graph. For sub-grouping, we applied the same methodology of rescaled, dimensionality reduction, and clustering to distinct dataset, usually confined to a specific type of cell. For each cluster, we employed the Wilcoxon Rank-Sum Test to identify significantly differentially expressed genes compared to the remaining clusters. SCINA and recognized marker genes were used to determine cell type.

**Differential expression analysis and Functional enrichment**

To identify DEGs (differential expression genes) between two distinct samples or clusters, we employed the Find Markers function within Seurat, using a likelihood ratio test. DEGs with a log2 fold change (log2FC) > 0.25 and a P values ≤ 0.05 were considered significantly differentially expressed. Concurrently, GO functional-enrichment analysis was performed to determine which DEGs were significantly enriched in GO terms and metabolic pathways, with a Bonferroni-corrected P value ≤0.05 relative to the transcriptome. GO functional enrichment analyses were conducted using Goatools (https://github.com/tanghaibao/Goatools).

**Establishment of diabetes model**

Two diabetes models were constructed using streptozotocin (STZ) and a high fat diet (HFD), respectively. Briefly, STZ was dissolved in a sterile sodium citrate solution with a pH of 4.5, 0.05 mol/L. Homozygous male C57BL/6J mice, 8 weeks of age, were divided into two groups. The diabetic group was administered 60mg/kg STZ for five consecutive days, while the control group received an equivalent volume of sodium citrate solution. Two weeks after the final STZ injection, the tail vein blood glucose was measured using a glucose meter (Accu-Chek, Roche Diagnostics GmbH, Mannheim, Germany). A postprandial blood glucose level > 16.7 mmol/L was classified as diabetes. The expression of related proteins in cardiac vascular endothelium was evaluated 12 weeks after the STZ injection.

Another diabetes model was established using HFD comprising 60% fat, 20% carbohydrate, and 20% protein (Trophic Animal Feed High-Tech Co., Nantong, Jiangsu, China). The model mice were started on HFD at 6 weeks of age, and their body weight and blood glucose were monitored every two weeks. A fasting blood glucose level >11.1 was considered a successful establishment of the model. The expression of associated proteins in cardiac vascular endothelium was evaluated after 12 weeks of HFD treatment.

**Histological analysis**

Masson trichrome staining was implemented according to the kit specifications (G1006, Servicebio, Wuhan, China). Upon thorough debridement and subsequent immersion in dye solution A overnight, the dissected specimens were subsequently cleaned using distilled water. The tissue slices then underwent an equal admixture treatment with dye solutions B and C for one minute under rigorous observation before undergoing another cleansing procedure with distilled water. Subsequently, dyes D was implemented for a duration of 6 minutes, dye E was utilized for one minute, and dye F for half a minute. Upon completion, these slices were rinsed meticulously and subsequently discerned using 1% acetic acid, followed by dehydration in two cycles of anhydrous ethanol. Lastly, they were rendered transparent via xylene for 5 minutes and sealed with neutral gum. The collagen fibers displayed various shades of blue ranging from pale to profound. Muscle fibers, cytoplasm, cellulose, and keratin exhibited a spectrum of colors spanning from white to reddish-purple. Red blood cells were distinctly visible as pink in color.

The periodic acid-silver methenamine (PASM) procedure is employed for precise quantification of the glomerular basement membrane thickness within the kidneys, as dictated by the kit protocols for PASM staining (G1059, Servicebio, Wuhan, China). Following paraffin section dehydration, specimens are impregnated with stain A overnight, washed extensively with purified water and then subjected to stain B incubation for 20 minutes. Integrate stain D, C, E into water to formulate the working solution. Heating this mixture to a temperature of 60 degrees Celsius for an interval of 20 minutes precedes its application onto the sections for a duration of 50 minutes. Subsequently, the sections are rinsed thrice with water and subjected to a five-minute treatment with stain F, followed by further rinsing and drying before encapsulation. The glomerular capsule basement membrane, glomerular capillary basement membrane, and renal tubular epithelial basement membrane within kidney tissues exhibit distinctive black pigmentation against a pink or orange backdrop.

Table S1. Primary antibodies used for immunoprecipitation **and** immunohistochemistry

| Antibody | Working dilutions | Catalog No. | Supplier |
| --- | --- | --- | --- |
| CD31 | ICC/IF: 1/100 | ab222783 | Abcam, Cambridge, UK |
| CD31 | ICC/IF: 1/200 | MA1-26196 | Invitrogen, CA, USA |
| Cfh | IF: 1/200 | sc-166613 | Santa Cruz, CO, USA |
| Ets1 | IF: 1/200 | ab307672 | Abcam, Cambridge, UK |
| Flag | IF: 1/200 | ab18230 | Abcam, Cambridge, UK |
| VCAM1 | IF: 1/200 | ab134047 | Abcam, Cambridge, UK |
| VE-cadherin | IF: 1/200 | 14-1441-82 | Invitrogen, CA, USA |
| F4/80 | ICC: 1/200 | #30325 | CST, MA, USA |

Table S2. Primer sequences for qPCR

| Mouse | Forward 5’ to 3’ | Reverse 5’ to 3’ |
| --- | --- | --- |
| TNF-α | CCCTCACACTCAGATCATCTTCT | GCTACGACGTGGGCTACAG |
| IL-1β | GCAACTGTTCCTGAACTCAACT | ATCTTTTGGGGTCCGTCAACT |
| IL-6 | TAGTCCTTCCTACCCCAATTTCC | TTGGTCCTTAGCCACTCCTTC |
| ICAM1 | GTGATGCTCAGGTATCCATCCA | CACAGTTCTCAAAGCACAGCG |
| β-actin | AACAGTCCGCCTAGAAGCAC | CGTTGACATCCGTAAAGACC |

Table S3. Body weight and random blood glucose in STZ-induced diabetic mice

| STZ | 0W | 2W | 4W | 6W | 8W | 10W | 12W |
| --- | --- | --- | --- | --- | --- | --- | --- |
| Body weight  （g） | 22.8±1.6 | 22.9±1.7 | 23.2±1.8 | 23.5±1.9 | 22.9±1.8 | 21.6±1.6 | 20.8±1.7 |
| Blood glucose（mmol/L） | 6.8±0.8 | 7.5±0.9 | 7.1±1.1 | 13.5±2.4 | 22.6±2.8 | 24.6±2.9 | 23.8±2.7 |

Table S4. Body weight and fasting blood-glucose in HFD-induced diabetic mice

| HFD | 0W | 2W | 4W | 6W | 8W | 10W | 12W |
| --- | --- | --- | --- | --- | --- | --- | --- |
| Body weight  （g） | 23.1±1.6 | 27.4±3.1 | 31.4±3.7 | 36.3±3.8 | 39.1±3.9 | 42.6±3.6 | 45.3±3.9 |
| Blood glucose（mmol/L） | 6.7±0.8 | 6.8±0.9 | 7.2±1.1 | 7.5±1.2 | 9.3±1.5 | 11.8±1.8 | 13.6±2.6 |


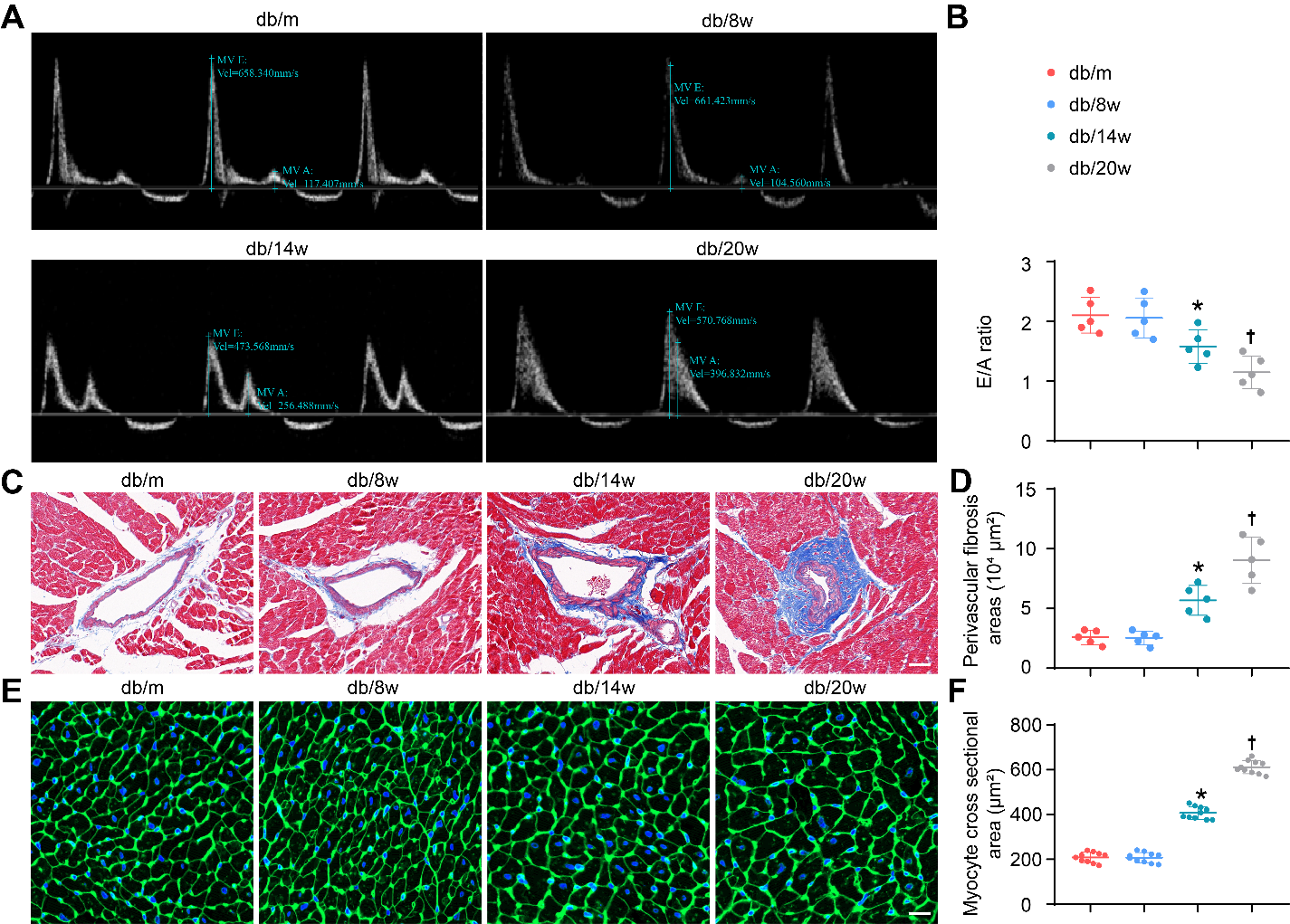


**Figure S1.** **Evaluation of cardiac injury.** (A-B) Representative Doppler echocardiographic images and quantification of ratio between early and late mitral diastolic waves (E/A ratio) (n=5). (C-D) Representative and quantified Masson's trichrome staining of perivascular tissues (n=5). Scale bars depict a length of 40 μm. (E-F) Representative images of wheat germ agglutinin staining and quantification of cardiomyocyte cross-sectional area in different groups (n=10). Scale bars depict a length of 25 μm. **P* < 0.05 vs. db/m; ^†^*P* < 0.05 vs. db/14w.


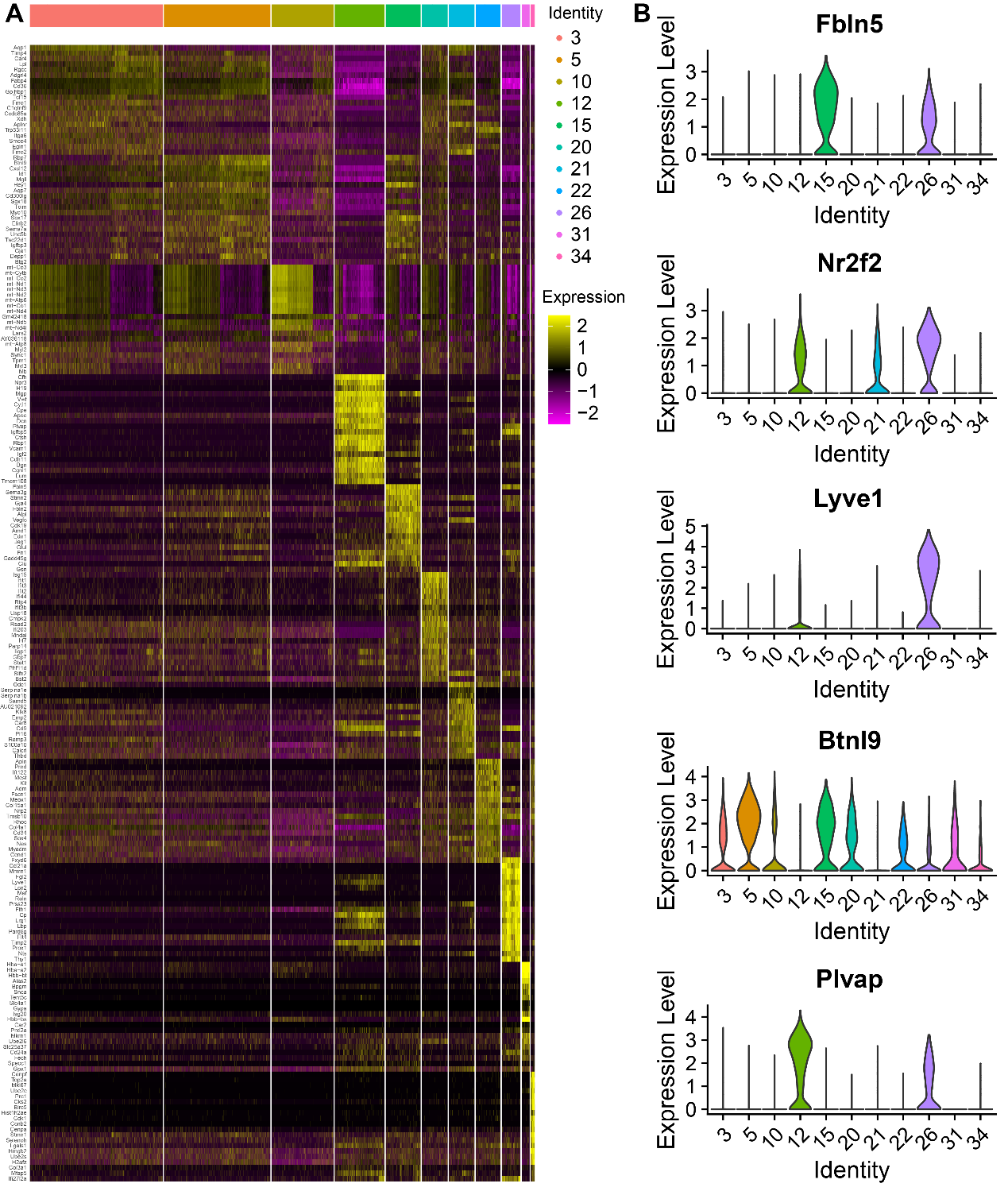


**Figure S2.** **Characterization assessment of individual cell cluster.** (A) Heat map displaying the top 20 differentially expressed genes in each cell cluster. (B) Representation of key genes defining diverse vascular types within each subcategory (arterial: Fbln5; venous: Nr2f2; Capillary: Btnl9; endocardial: Plvap; lymphatic: Lyve1).


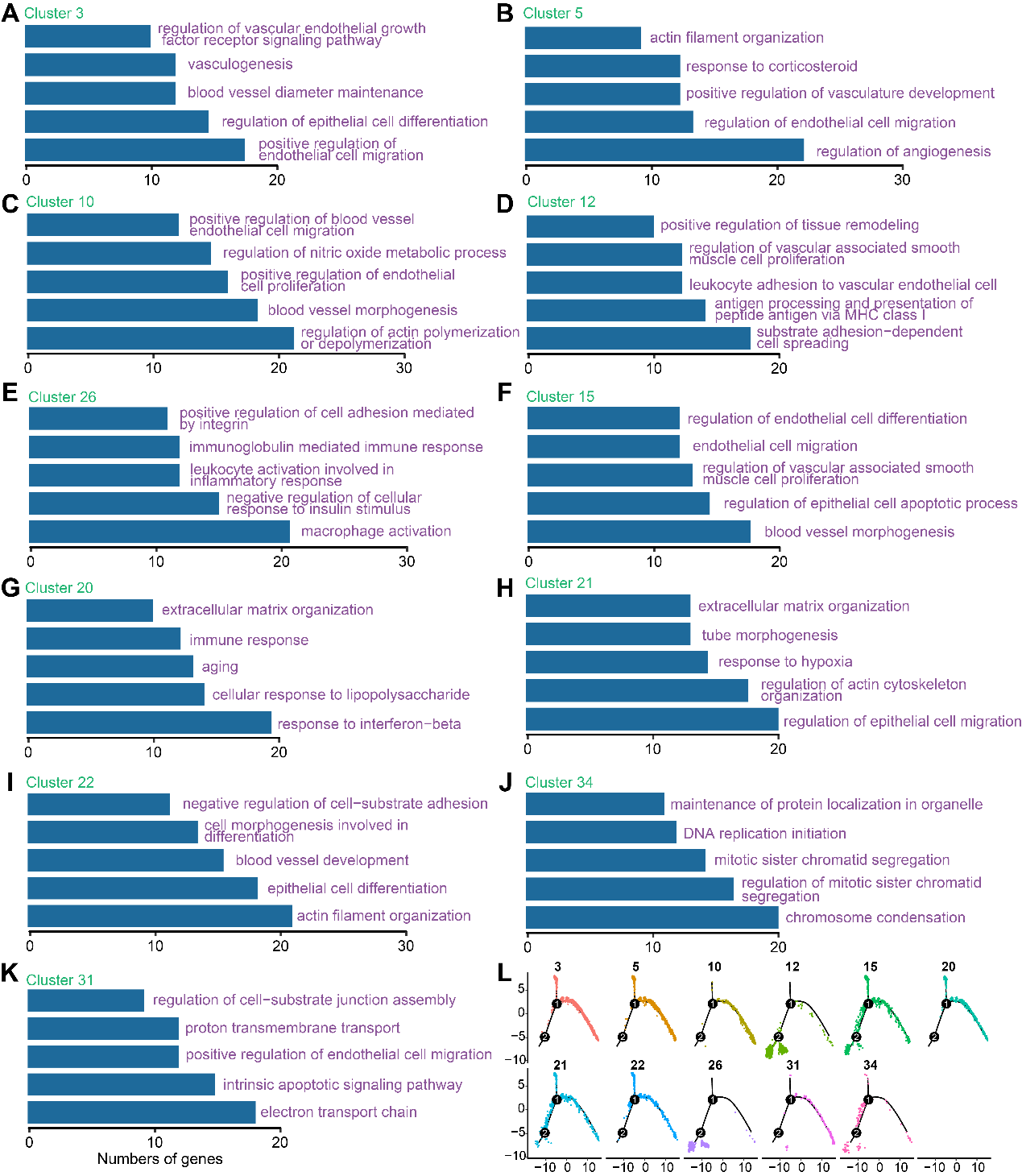


**Figure S3. Monocle analysis and functional annotation of cell clusters.** (A-K) GO analysis of genes differentially expressed in each cluster. (L) Monocle analyses revealing the positions of distinct clusters along pseudotime trajectories, the minute dots of varying hues depict the corresponding cell clusters, and the 1 and 2 at the branches signify the two nodes when the functionality of cell clusters is transformed.


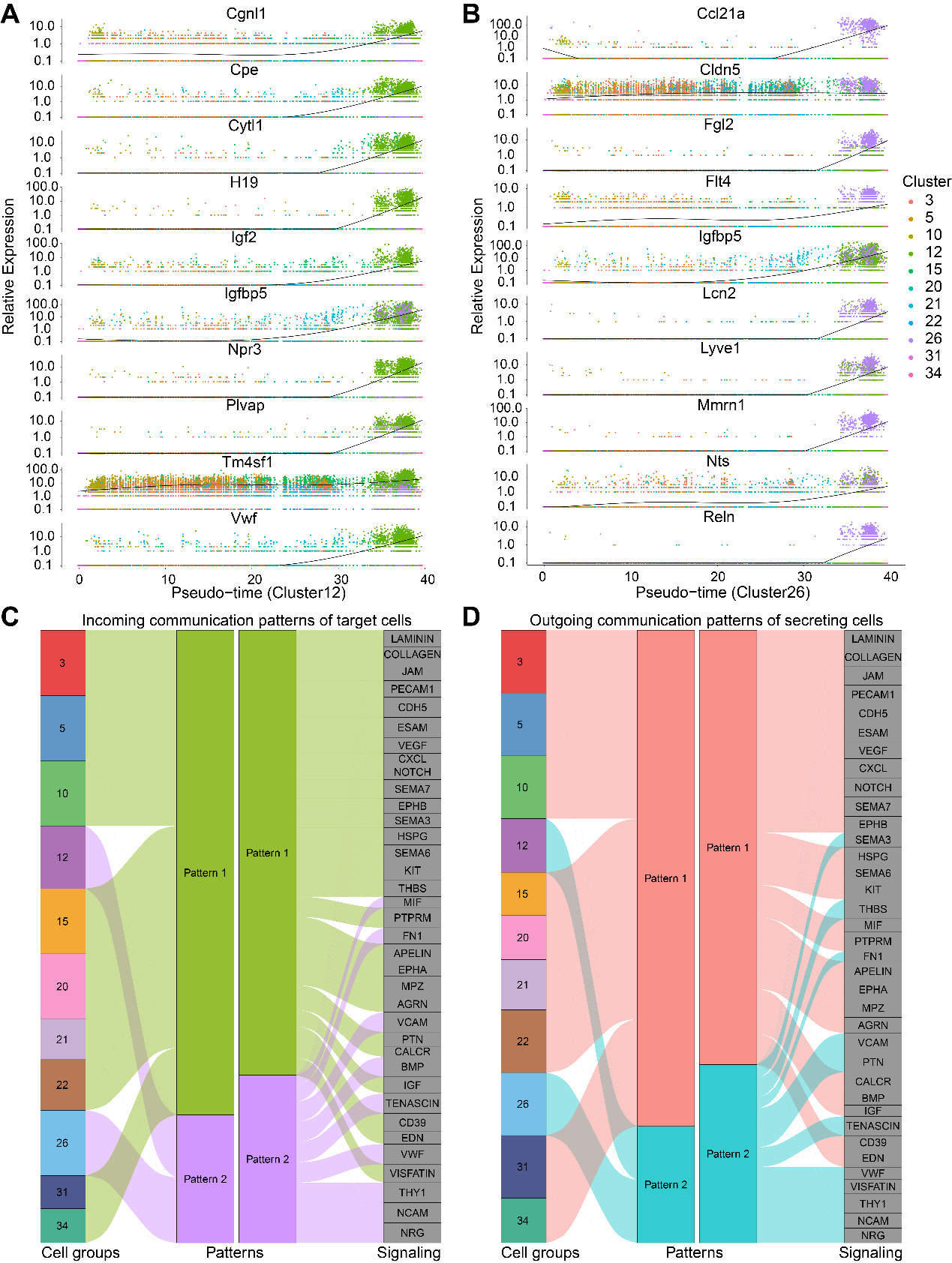


**Figure S4.** **Monocle analysis and communication patterns of cell clusters.** (A) Top 10 differentially expressed genes of cluster 12 along pseudotime trajectories. (B) Top 10 differentially expressed genes of cluster 26 along pseudotime trajectories. The point color was determined according to the cluster, Igfbp5 is among the top 10 differentially expressed genes of both 12 and 26 clusters. (C) Incoming communication patterns of target cells. (D) Outgoing communication patterns of target cells.


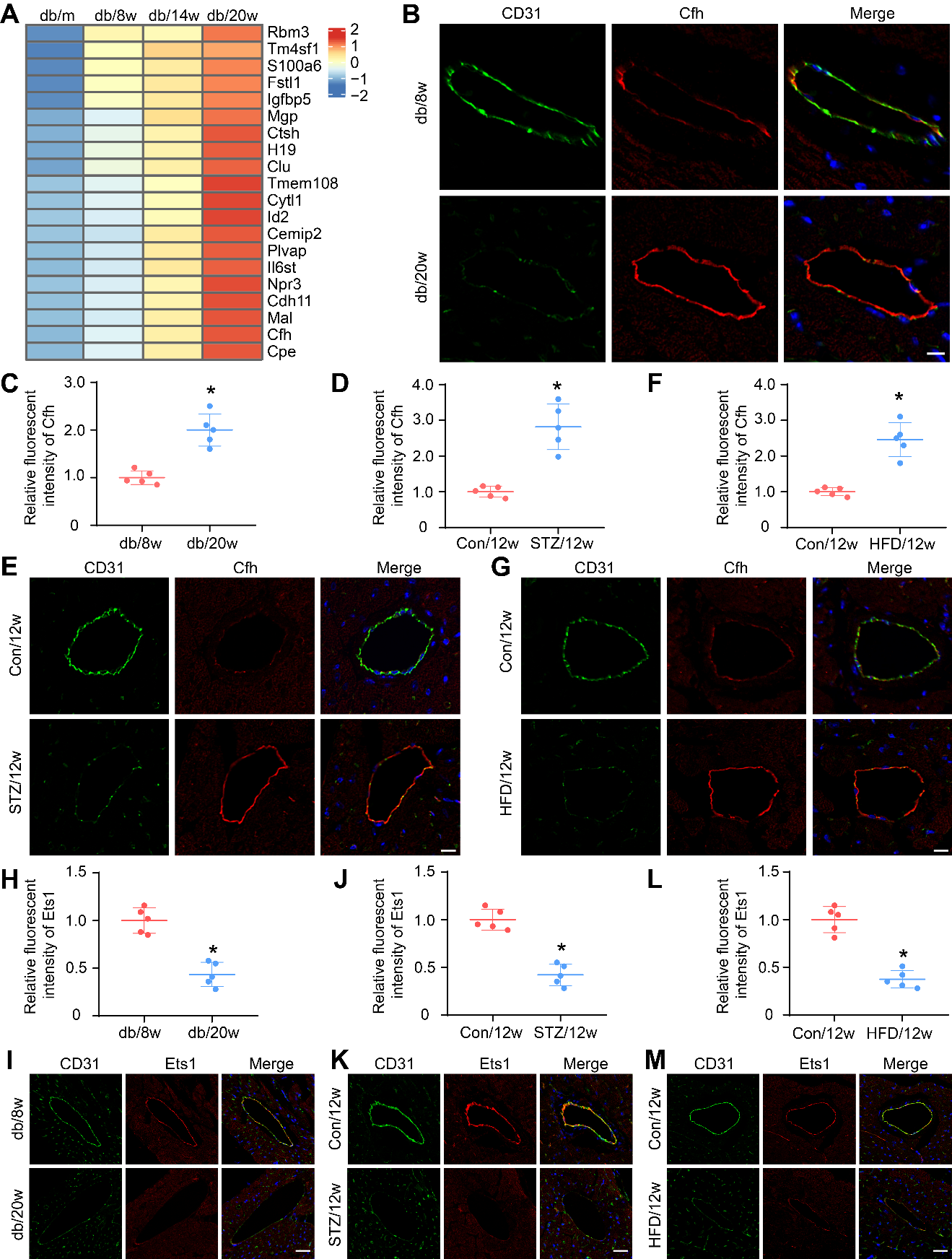


**Figure S5.** **Assessment of expression level of relevant proteins.** (A) Twenty genes upregulated in ECs throughout the progression of diabetes. (B) The protein level of Cfh was assessed through immunofluorescence staining in cardiac vasculature of db/db mice at various stages (n=5). Scale bars = 10 μm. (C) Quantification of the fluorescent intensity of Cfh immunofluorescence staining in db/db mice from different group. **P* < 0.05 vs. db/8w. (D-E) Representative and quantified immunofluorescence staining of Cfh in STZ-treated and control mice (n=5). Scale bars depict a length of 10 μm. **P* < 0.05 vs. Con/12w. (F-G) Representative and quantified immunofluorescence staining of Cfh in HFD-treated and control mice (n=5). Scale bars depict a length of 10 μm. **P* < 0.05 vs. Con/12w. (H-I) Representative and quantified immunofluorescence staining of Ets1 in db/db mice from different group (n=5). Scale bars depict a length of 30 μm. **P* < 0.05 vs. db/8w. (J-K) Representative and quantified immunofluorescence staining of Ets1 in STZ-treated and control mice (n=5). Scale bars depict a length of 30 μm. **P* < 0.05 vs. Con/12w. (L-M) Representative and quantified immunofluorescence staining of Ets1 in HFD-treated and control mice (n=5). Scale bars depict a length of 30 μm. **P* < 0.05 vs. Con/12w.


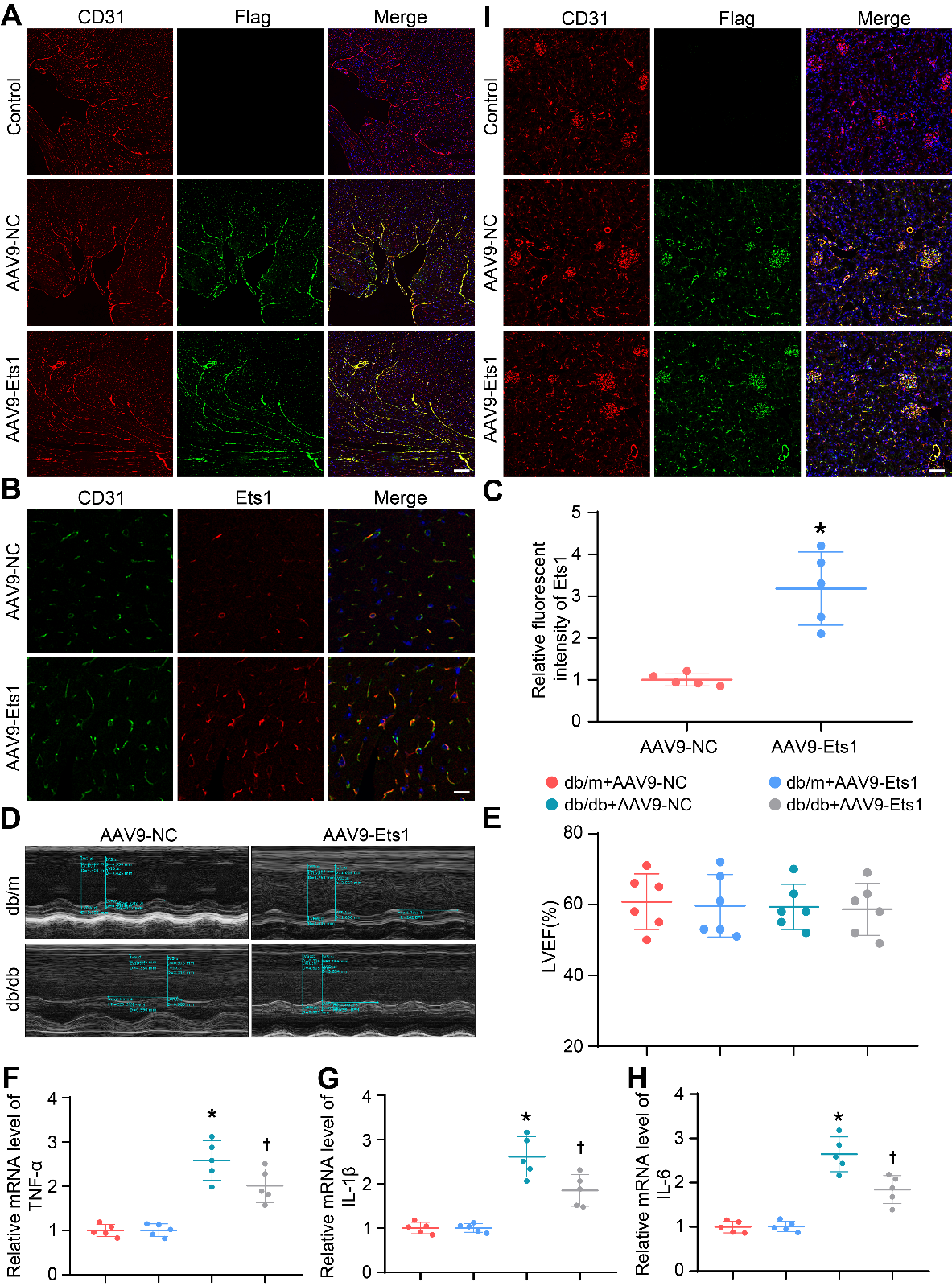


**Figure S6. Assessment of expression level of relevant proteins.** (A) AAV9 transfection efficiency was detected using immunofluorescence staining of Flag and CD31 in AAV9-NC or AAV9-Ets1 infected heart. Scale bars depict a length of 150 μm. (B-C) Representative and quantified immunofluorescence staining of Ets1 (n=5). Scale bars depict a length of 10 μm. **P* < 0.05 vs. AAV9-NC. (D-E) Representative Doppler echocardiography images and quantification of the ratio between the early and late mitral diastolic waves (E/A ratio) (n=6). (F-H) The relative mRNA level of TNF-α, IL-1β, and IL-6 in hearts (n=5). *P < 0.05 vs. db/m+AAV-NC; **^†^**P < 0.05 vs. db/db+AAV-NC. (I) AAV9 transfection efficiency was detected using immunofluorescence staining of Flag and CD31 in AAV9-NC or AAV9-Ets1 infected kidney. Scale bars depict a length of 100 μm.


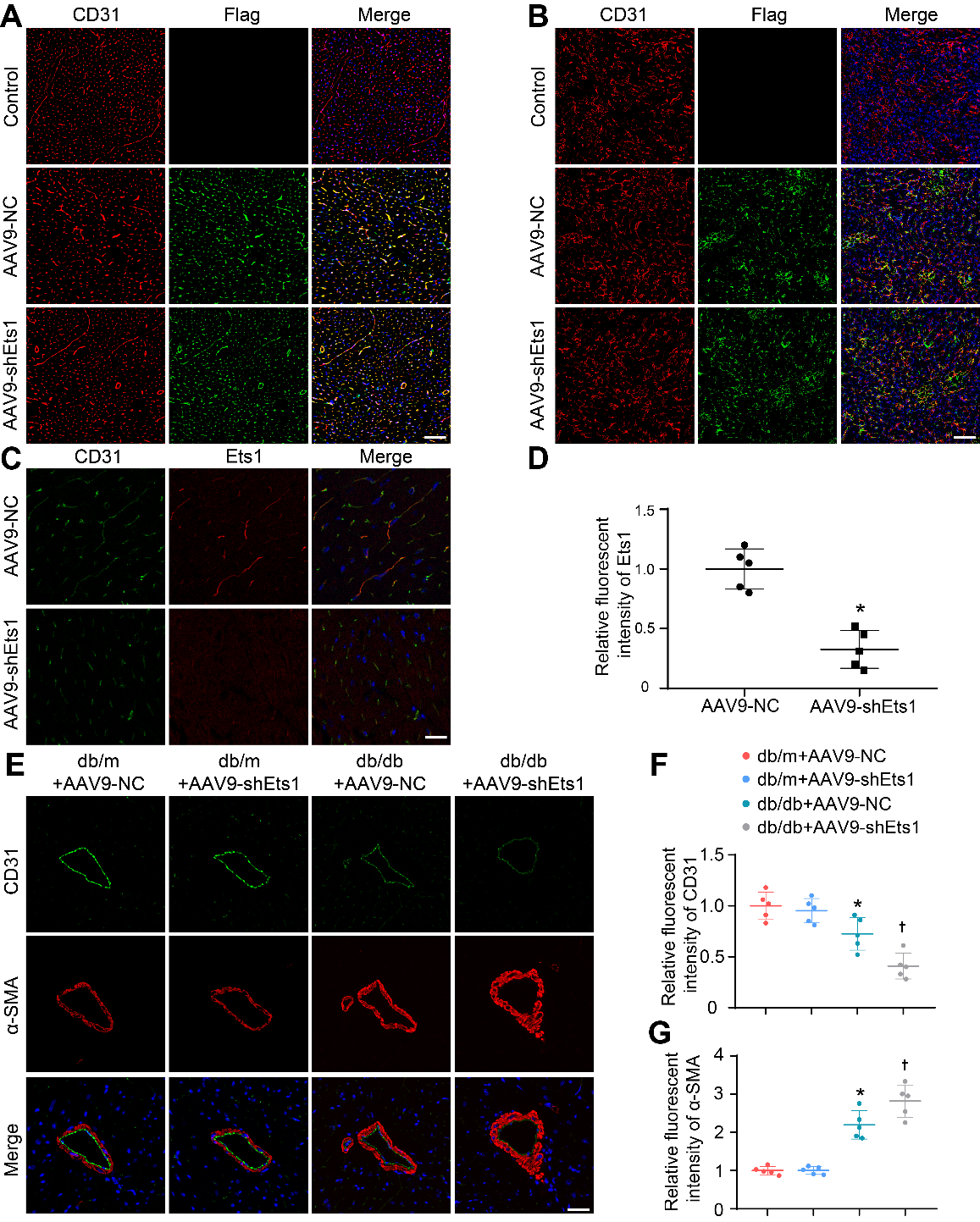


**Figure S7. Ets1 knockdown in endothelial cells exacerbates cardiac and renal injury in db/db mice.** (A) AAV9 transfection efficiency was detected using immunofluorescence staining of Flag and CD31 in AAV9-NC or AAV9-shEts1 infected heart. Scale bars depict a length of 100 μm. (B) AAV9 transfection efficiency was detected using immunofluorescence staining of Flag and CD31 in AAV9-NC or AAV9-shEts1 infected kidney. Scale bars depict a length of 100 μm. (C-D) Representative and quantified immunofluorescence staining of Ets1 (n=5). Scale bars depict a length of 10 μm. **P* < 0.05 vs. AAV9-NC.

(E-G) Representative and quantified immunofluorescence staining of CD31 and α-SMA (n=5). The scale bars depict a length of 25 μm. **P* < 0.05 vs. db/m+AAV-NC; ^†^*P* < 0.05 vs. db/db+AAV-NC.


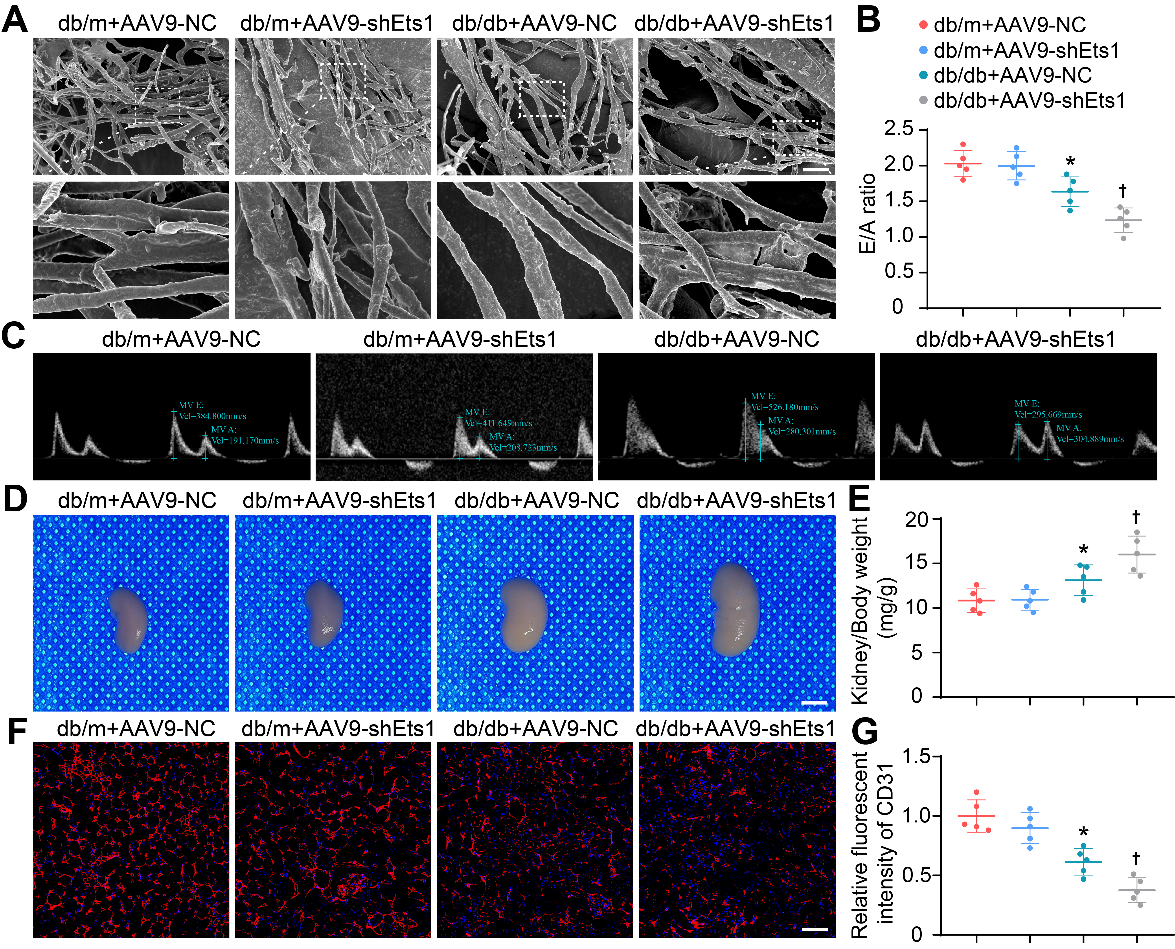


**Figure S8. Ets1 knockdown in endothelial cells exacerbates cardiac and renal injury in db/db mice.**

(A) Representative scanning electron micrographs of cardiac vascular corrosion in various groups. Scale bars depict a length of 10 μm. (B-C) Representative Doppler echocardiography images and quantification of ratio between early and late mitral diastolic waves (E/A ratio) (n=5). (D-E) Representative kidney images and quantification of kidney/body weight in various groups. (F-G) Representative and quantified immunohistochemical staining of CD31 of kidney in different groups (n=5). **P* < 0.05 vs. db/m+AAV-NC; ^†^*P* < 0.05 vs. db/db+AAV-NC.
